# Profiling of Luteal Transcriptome during Prostaglandin F2-Alpha Treatment in Buffalo Cows: Analysis of Signaling Pathways Associated with Luteolysis

**DOI:** 10.1371/journal.pone.0104127

**Published:** 2014-08-07

**Authors:** Kunal B. Shah, Sudeshna Tripathy, Hepziba Suganthi, Medhamurthy Rudraiah

**Affiliations:** Department of Molecular Reproduction, Development and Genetics, Indian Institute of Science, Bangalore, India; Justus-Liebig-Universität, Germany

## Abstract

In several species including the buffalo cow, prostaglandin (PG) F_2α_ is the key molecule responsible for regression of corpus luteum (CL). Experiments were carried out to characterize gene expression changes in the CL tissue at various time points after administration of luteolytic dose of PGF_2α_ in buffalo cows. Circulating progesterone levels decreased within 1 h of PGF_2α_ treatment and evidence of apoptosis was demonstrable at 18 h post treatment. Microarray analysis indicated expression changes in several of immediate early genes and transcription factors within 3 h of treatment. Also, changes in expression of genes associated with cell to cell signaling, cytokine signaling, steroidogenesis, PG synthesis and apoptosis were observed. Analysis of various components of LH/CGR signaling in CL tissues indicated decreased LH/CGR protein expression, pCREB levels and PKA activity post PGF_2α_ treatment. The novel finding of this study is the down regulation of CYP19A1 gene expression accompanied by decrease in expression of E_2_ receptors and circulating and intra luteal E_2_ post PGF_2α_ treatment. Mining of microarray data revealed several differentially expressed E_2_ responsive genes. Since CYP19A1 gene expression is low in the bovine CL, mining of microarray data of PGF_2α_-treated macaques, the species with high luteal CYP19A1 expression, showed good correlation between differentially expressed E_2_ responsive genes between both the species. Taken together, the results of this study suggest that PGF_2α_ interferes with luteotrophic signaling, impairs intra-luteal E_2_ levels and regulates various signaling pathways before the effects on structural luteolysis are manifest.

## Introduction

Corpus luteum (CL) is a transient endocrine structure formed from the remnants of the ovulating follicle. Through secretion of progesterone (P_4_), it plays a pivotal role in the control of reproduction in mammals. During non-pregnant cycles, regression of CL precedes initiation of new reproductive cycle to allow reovulation and another chance for conception to occur [1]. The structure and function of CL are controlled by intricate interplay between luteotrophic and luteolytic factors. In several species including bovines, the influence of lytic factors dominate over luteotropic factors in the control of luteal function. Of the many factors, prostaglandin (PG) F_2α_ molecule is recognized as the physiological luteolysin responsible for luteal regression in mammals [2]–[4].

PGF_2α_ or its synthetic analogues initiate a complex cascade of events within the CL leading to inhibition of steroidogenesis resulting in rapid fall in circulating P_4_ levels (functional luteolysis) [5]–[7] and initiation of apoptosis (structural regression) in the bovine species [6], . In several species, many of the endocrine, biochemical and morphological events observed during PGF_2α_-induced luteolysis have been reported to be broadly similar [11]–[20]. Disruption of transport of cholesterol due to inhibition of StAR expression has been implicated as the cause of inhibition of steroidogenesis after PGF_2α_ treatment under both *in vivo* and *in vitro* conditions [21], [22]. However, in a more recent study, an initial increase in StAR expression post intrauterine PGF_2α_ treatment has been observed [23], suggesting factors other than StAR are responsible for inhibition of steroidogenesis. Studies on molecular analysis of structural regression of bovine CL leading to apoptosis has previously been reported by others [24], [25] and us [6], [9], but the actual mechanisms responsible for functional luteolysis has not been systematically examined. Recent studies on transcriptome analysis of CL tissues have expanded our understanding of effects of PGF_2α_ during luteolysis or refractory state of CL to PGF_2α_ effects [26]–[28].

In contrast to a well-established role for pituitary LH in the control of CL function in primates and rodents [29], [30], the role of LH in the regulation of CL function in bovines has not been clearly established. In cattle, an earlier study had suggested a role for LH in CL function [31], while other studies have suggested a minor role for LH especially during pregnancy [32]–[34]. To understand mechanisms involved in the initial loss of CL function in response to PGF_2α_ treatment, it is essential to examine molecules downstream of LH/CG receptor (LH/CGR) activation during PGF_2α_-induced luteolysis. The role of intraluteal molecules during luteolysis forms another crucial area for examining the PGF_2α_ actions during the initial period of PGF_2α_ treatment [35]. In bovines, even though expression of CYP19A1 that codes for aromatase enzyme responsible for converting androgens to estrogens, gets down regulated after ovulation, its expression is restored albeit at low levels in the developed CL and is never the less responsible for low levels of intraluteal estradiol-17β (E_2_) [36] in the buffalo cow. Since luteal tissue expresses both α and β receptors [37], it remains to be determined whether biosynthesis of E_2_ and its receptor signaling is disrupted following PGF_2α_ treatment contributes to the loss of luteal function. Interestingly, E_2_ is the primary luteotrophic hormone in rabbits and experimental withdrawal of E_2_ has been shown to inhibit luteal function and activate apoptotic pathways [38], [39].

In view of the above, the following objectives were addressed in the present study: 1) analysis of global gene expression changes in CL of buffalo cows administered with luteolytic dose of PGF_2α_ treatment, 2) examination of effects of PGF_2α_ on molecules downstream of LH receptor signaling and 3) examination of the effects of PGF_2α_ on CYP19A1 expression, E_2_ biosynthesis and expression of E_2_ responsive genes in the CL.

## Materials and Methods

### Ethics Statement

All procedures in animals were approved by the Institutional Animal Ethics Committee, Indian Institute of Science, Bangalore, India.

### Reagents

Juramate (Cloprostenol sodium, a synthetic analogue of PGF_2α_) was purchased from Jurox Pty Ltd, Rutherford, Australia. P_4_ (GDN#337) and E_2_ (GDN#244) antisera was kindly provided by Prof. G. D. Niswender, Colorado State University, Fort Collins, CO. The enzyme deoxynucleotidyl transferase was purchased from Amersham Pharmacia Biotech Asia Pacific (Bangalore, India). Moloney murine leukemia virus reverse transcriptase (MMuLV RT) enzyme and 100 bp DNA ladder were obtained from MBI Fermentas GmbH (St. Leon-Rot, Germany). Oligonucleotide and Oligo dT primers were synthesized by Sigma-Genosys (Bangalore, India). DyNAzyme II DNA polymerase was purchased from Finnzymes (Espoo, Finland) and dNTPs were procured from Eppendorf, (Hamburg, Germany). Power SYBR Green PCR master mix was obtained from Applied Biosystems (Foster City, CA). Labeled α-^32^P dCTP (LCP 103, 3000 Ci/mmole) was procured from BRIT (Hyderabad, India). Random primer extension labeling kit (#KT04) was procured from Bangalore Genei (Bangalore, India). Nylon (Gene screen plus) and PVDF membranes were purchased from NEN life Sciences, (Boston, MA). Polyclonal antibodies specific to LH/CGR (H-50: sc-25828), SF-1 (H-60: sc-28740) and LRH-1 (H-75: sc-25389) were purchased from Santa Cruz Biotechnology Inc (Santa Cruz city, CA). Antibody for β-actin (#A3854) was purchased from Sigma-Aldrich Co. (St.Louis, MO). pCREB (#9191), CREB (#9192), pAkt (#4060), Akt (#9272), pFKHR (#9464), FKHR (#9462), pPI3k p85 (#4228), PI3k p85 (#4257) and horse radish peroxidase labeled goat anti-rabbit IgG and ECL chemiluminescence kit were purchased from Cell Signaling Technology (Beverly, MA). Antibodies for Bax (44–62: 196820) and Bcl-2 (4–21: 197207) were purchased from Calbiochem-Novabiochem Corporation (La Jolla, CA). StAR antibody was a gift from Professor D. M. Stocco (Texas Tech University Health Sciences Center, Lubbock, TX). All other reagents were purchased from Sigma-Aldrich Co. (Bangalore, India) and Life Technologies (Carlsbad, CA) or sourced from local distributors.

### Experimental protocol, blood and CL collection schedule

Non-lactating buffalo cows (*Bubalus bubalis*, Surthi breed) aged 5–6 years, with a history of normal cyclicity were monitored daily for onset of behavioral signs of estrus such as bellowing, restlessness and mucous discharge from the vulva. The estrous cycles were synchronized with two injections of PGF_2α_ and the day of onset of estrus was designated as day 0 of estrous cycle. To verify the presence of functional CL, blood samples were collected on days 3 to 7 of the cycle for monitoring circulating P_4_ concentration. In this experiment, Juramate (500 µg i.m.) was administered on day 11 of estrous cycle and blood samples (n = 5–6 animals) were collected immediately before and at various time points post PGF_2α_ treatment. CL was collected at slaughter from untreated control animals (0 h), as well as from animals at 1, 2, 3, 6 and 18 h post PGF_2α_ injection (n = 3–4 animals/time point). For serum E_2_ estimation, blood sampling has also been done at earliest time points, i.e. at 1 and 2 h post PGF_2α_ treatment. CL tissues were also collected at varied points throughout an estrous cycle with day 5, 11, 14, 17 and 20 CL being designated as early (E), mid (M), mid-late (ML), late (L) and very late (VL) CL, respectively. Ovaries containing CL were collected and washed in sterile ice cold PBS and transferred into Dulbecco's Modified Eagles Medium supplemented with penicillin (500 U/ml) and streptomycin (50 µg/ml) and transported to the laboratory on ice within 30 min of collection. Following retrieval of CL from the ovary, CL was cut into ∼50 mg pieces and 3–4 pieces were placed in sterile cryovial and flash frozen in liquid nitrogen before storing at −70 °C for various analyses. The progesterone analysis data and a portion of CL tissue used for different analysis have been reported recently [40].

### Hormone assays

For the measurement of luteal P_4_ and E_2_, wet weight of one of the CL pieces was determined before homogenization in a known volume of 1X PBS solution. Undiluted CL tissue lysate (E_2_) or at a dilution of 1∶500 (P_4_) was used in the assay. Hormone extraction from lysate was carried out using diethyl ether and reconstituted in GPBS at 37°C for 1 hour in an incubator shaker. Luteal and serum P_4_ and E_2_ concentrations were determined by radioimmunoassay method as reported previously [41]. The sensitivity of the assay was 10 pg/tube and 3.9 pg/tube for P_4_ and E_2_, respectively and the inter- and intra- assay coefficients of variation were <10%.

### Isolation of genomic DNA and fragmentation analysis

Genomic DNA was extracted from individual CL using standard phenol-chloroform extraction method. The isolated genomic DNA was precipitated, dissolved and quantitated spectrophotometrically for fragmentation analysis as described elsewhere [6]. Briefly, genomic DNA from different CL tissue samples were analyzed for quantitation of low molecular weight DNA fragmentation using protocol described previously [42], [43] with few modifications. Genomic DNA (4 µg) was labeled with 50 µCi of [α^32^P] dCTP by incubation at 37°C for 1 h with 10 units of terminal deoxynucleotidyl transferase enzyme. The DNA samples were then resolved on a 2% agarose gel and transferred onto a nylon transfer membrane using the alkaline (0.4 M NaOH and 1 M NaCl) buffer with capillary blotting transfer method for 16 h. The membranes post transfer were cross linked using 1200 µJoules of UV for 1 min in Stratalinker. The membranes were covered with plastic wrap and were exposed to an intensifying screen at room temperature and the low molecular weight labeled DNA signals were quantitated densitometrically using a PhosphorImager (Typhoon 9210; Amersham Biosciences) with exposure of 6 h or longer.

### RNA isolation

Total RNA was extracted from control and PGF_2α_ treated CL tissues using TRI Reagent according to the procedure as reported previously [41]. RNA was quantitated spectrophotometrically using ND-1000 (NanoDrop, Thermo Scientific, Wilmington, DE). The quality and quantity of RNA were determined by electrophoresis on a 2% (w/v) formaldehyde agarose gel along with RNA samples of known concentration, and A_260_: A_280_ ratio was >1.8. Additionally, the quality of RNA samples was assessed using Agilent Bioanalyzer 2100 and RNA samples with RNA integrity number ≥9 were used for microarray analysis.

### Quantitative real time PCR (qPCR)

The qPCR analysis was carried out as described previously from the laboratory [44]. The cDNA samples equivalent to 10 ng of total RNA were subjected to validation analysis on Applied Biosystems 7500 Fast Real Time PCR system with SDS v 1.4 program employing Power SYBR green 2X PCR master mix. The details of primers employed along with the annealing temperature and expected product size are provided in Table S1. Analysis of expression of each gene included a no template control (NTC) and generation of a dissociation curve. Expression level of individual gene was normalized by using L19 expression level as calibrator (internal control) for each cDNA sample. The relative expression of each gene was determined using the ΔΔC_t_ method which calculates the fold change in gene expression using the formula: Fold change  = 2^−ΔΔCt^, where C_t_ =  Threshold cycle i.e. the cycle number at which the relative fluorescence of test samples increases above the background fluorescence and ΔΔC_t_ =  [C_t_ gene of interest (unknown sample) - C_t_ of L19 (unknown sample)] - [C_t_ gene of interest (calibrator sample) - C_t_ of L19 (calibrator sample)]. PCR for each sample was set up in duplicates and the average C_t_ value was used in the ΔΔC_t_ equation.

### Immunoblot analysis

Immunoblot analysis of the total protein lysates from CL tissues was carried out as per the procedure reported previously [6].

### Protein kinase A assays

Protein kinase A assays were performed according to the manufacturer's instructions provided with the kit and as described in detail previously [45].

### Microarray target preparation and hybridization

Transcriptome analysis of CL tissues was performed on GeneChip Bovine Genome Array (Affymetrix, Santa Clara, CA) that contains 24,072 probe sets representing ∼23,000 bovine transcripts and few housekeeping/control genes. Use of heterologous array hybridization screening for gene expression changes of closely related species has been validated for many species [46]. Affymetrix Bovine GeneChip platform is very efficient and can be easily extended to other species for which genetic sequences are available [47], [48]. The water buffalo and domestic cattle, both belonging to the Bovidae family, are closely related. Moreover, use of robust multiarray average (RMA) algorithm for background correction, normalization across arrays and summarization is well suited for cross species microarray analysis, since the software considers hybridization from perfect match probes and not the mismatch probes [47]. More recently, microarray analysis has been performed on buffalo granulosa cells employing GeneChip Bovine Genome Array and analysis data indicated excellent hybridization [49]. The entire procedure of microarray target preparation, hybridization and scanning were performed at Center for Integrated Biosystems, Utah State University, Utah by employing standard Affymetrix protocols (Chip Expression Analysis Technical Manual rev. 3, 2001). Further, scanning was performed using Affymetrix Genearray Scanner 3000 series.

### Analysis of microarray data

Total RNA isolated from control and PGF_2α_-treated CL tissue was labeled and hybridized to Affymetrix GeneChip Bovine Genome Arrays as per the manufacturer's recommendations. 3 Affymetrix GeneChip Bovine Genome Arrays were used for RNA from individual CL as biological replicates (n = 3/time point; 0, 3, 6 and 18 h post PGF_2α_ treatment). A total of 12 arrays were used. A detailed description of procedures and subsequent generation of processed image files of microarray analysis have been reported previously [44], [49]. The microarray procedure and the data analysis were performed as per Minimum Information About Microarray Experiments (MIAME) compliance. The raw data and the completed analysis of microarray data files have been deposited at NCBI's Gene Expression Omnibus, http://www.ncbi.nlm.nih.gov/geo/query/acc.cgi?acc=GSE27961 and are accessible through GEO series accession number GSE27961. The processed image files (.cel) were normalized across arrays using RMA software [50] and log-transformed (base 2), which allowed direct comparison of probe set values between all samples used in the experiment normalization. GeneSifter (VizX Labs; Seattle, WA, data not shown), GeneSpring GX (Agilent Technologies, Inc. Santa Clara, CA) and R/Bioconductor (FHCRC labs; Seattle, WA) microarray expression analysis softwares were used to identify differentially expressed transcripts. The differential expression of genes was calculated by averaging the normalized samples and performing a pairwise analysis. Statistical significance for differentially expressed genes among control and treatment groups was determined using Student's t-test (two tail, unpaired) employing Benjamini and Hochberg correction factor for false discovery rate [51]. As many of the original annotations for the Affymetrix Bovine Genome Chip have been reported to be erroneous [52], a computational tool, AffyProbeMiner, was employed to redefine the chip definition files taking into account the most recent genome sequence information [53]. AffyProbeMiner analysis results suggested that the original Affymetrix gene annotation were not compromised and the identified differentially expressed genes were transcript consistent and did not hybridize to multiple transcripts. Analysis of the data by Bioconductor analysis tool employing ≥2 fold change cut-off and statistical filters provided a number of differentially expressed genes. The linear regression analysis was performed on fold change expression values for selected differentially expressed genes obtained from qPCR and microarray analyses and a statistically significant (p<0.05) correlation between the two analyses was determined as reported previously [44], [49]. The list of top 15 differentially expressed (both up- and down-regulated) genes at different time points post PGF_2α_ administration are represented in Table S2, S3, S4, S5, S6, S7. The differentially expressed genes were clustered by hierarchy analysis by GeneSpring analysis for all the probe sets at each time point post PGF_2α_ administration and represented as dendrograms or heat map in Figure S1. Further, to examine the molecular function and genetic networks, the microarray data was analyzed using Ingenuity Pathways Analysis tool (IPA version 8.7, Ingenuity Systems Inc., Redwood City, CA, USA; http://www.ingenuity.com), a web-based software application that enables identification of biological mechanisms, pathways, and functions from the differentially expressed genes.

### Statistical analysis

The data were expressed as mean ± SEM. Statistical evaluation of mean differences of E_2_ and P_4_, protein blots and qPCR fold change in expression of genes among different treatment groups were analyzed using one-way ANOVA test followed by the Newman-Keuls multiple comparison tests (PRISM GraphPad; GraphPad Software Inc., San Diego, CA). A p-value of <0.05 was considered to be significant.

## Results

### Effects of PGF_2α_ on circulating P_4_, StAR expression and biochemical integrity of DNA (DNA laddering) in the CL tissue

Following administration of PGF_2α_, circulating P_4_ levels declined significantly within 1 h and continued to be lower at 2, 3, 6 and 18 h time points examined (p<0.05; Figure S2A). The luteal P_4_ level too declined significantly by 3 h and further declined by 6 and 18 h post PGF_2α_ treatment (p<0.05; Figure S2A). Analysis of StAR protein pattern paralleled P_4_ decline at all-time points examined (Figure S2B). Analysis of low molecular weight DNA fragments indicated that although a slight increase in low molecular weight fragments could be visualized in CL tissues at 6 h post PGF_2α_ treatment compared to untreated CL tissue, but a clear increase of laddering pattern of DNA was observed in CL tissues collected from animals, 18 h post PGF_2α_ treatment (Figure S2C). These results verified that CL tissue underwent a time related rapid functional loss followed by evidence of structural loss after initiation of PGF_2α_ treatment.

### Genome wide analysis of differentially expressed genes in the CL of buffalo cow post PGF_2α_ treatment

The mechanism of PGF_2α_-induced luteolysis is a complex process involving expression changes in genes associated with regulation of steroidogenesis, apoptosis, expression changes of gene products of several factors such as enzymes involved in PGF_2α_ biosynthesis and cross talk with other signaling pathways that include luteotrophic factors. In the present study, efforts were made to identify temporal changes in the profile of transcriptome of CL tissue in response to PGF_2α_ treatment. The microarray analysis provided a set of differentially expressed genes based on various high stringency statistical filters such as a t-test with p<0.05 and multiple hypothesis testing (Benjamini and Hochberg comparison test) to eliminate the false positives. Further, the differentially expressed genes that passed the statistical filters were classified based on whether the identified differentially expressed gene was present in all the samples examined. Another parameter employed was a sliding scale fold change cut-off filter of ≥2 (except in identification of E_2_ target genes, in which changes <1.5 fold was considered for analysis) with some degree of confidence in our candidate list to narrow down the list.

**Figure 1:**
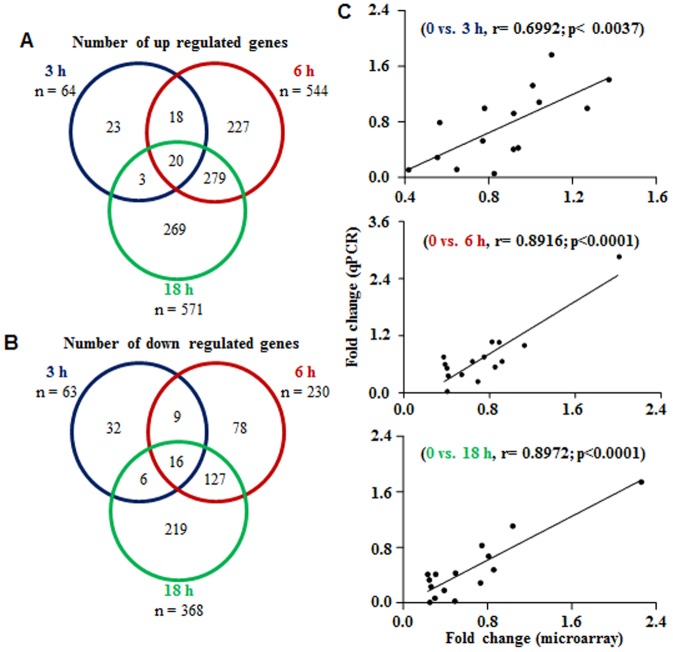
Schematic representation of differentially expressed genes in the CL post PGF_2α_ treatment. (A and B) Venn diagrams representing the number of differentially expressed genes identified after microarray analysis of CL tissues collected before (0 h) and at different time points post PGF_2α_ treatment. Data analyzed by Bioconductor analysis tool employing ≥2 fold change cut-off and statistical filters with Benjamini and Hochberg correction factor for false discovery rate. The comparison of total number of differentially expressed up (A) and down (B) regulated genes found common between 0 vs. 3 h (blue circle) and 0 vs. 6 h (red circle), as well as between 0 vs.18 h (green circle) post PGF_2α_ treatment are presented. (C) Correlation analysis between expression ratios obtained from microarray and qPCR analyses of few genes post PGF_2α_ treatment (n = 15 genes/time point). Linear regression analysis was performed for selected differentially expressed genes using qPCR fold change in expression values (2^−ΔΔCT^; Y-axis) with fold change expression values obtained by microarray analysis (X-axis). The r value, correlation coefficient, generated for the theoretical line of best fit (represented as solid line in each panel) and the p value indicate the significance of the correlation as determined by ‘F’ test.

The whole transcriptome analysis data presented as Venn diagrams in Figure 1A and B revealed a total of 127, 774 and 939 genes differentially expressed in CL tissues collected at 3, 6 and 18 h post PGF_2α_ treatment, respectively. Also, the distribution of nearly 40 differentially expressed genes which were common across different time points post PGF_2α_ treatment were identified as up regulated (20 genes), down regulated (16 genes) or up regulated at one time point, but down regulated at another time points (4 genes) are shown in the Venn diagrams (Figure 1A&B). It was observed that most cluster of genes appeared to be modulated in a synchronous or synergistic way following PGF_2α_ treatment, i.e. a higher percentage of the genes regulated at the 3 h time point had a similar regulation at 6 and 18 h time points.

### qPCR validation of differentially expressed genes from the microarray analysis data

**Figure 2:**
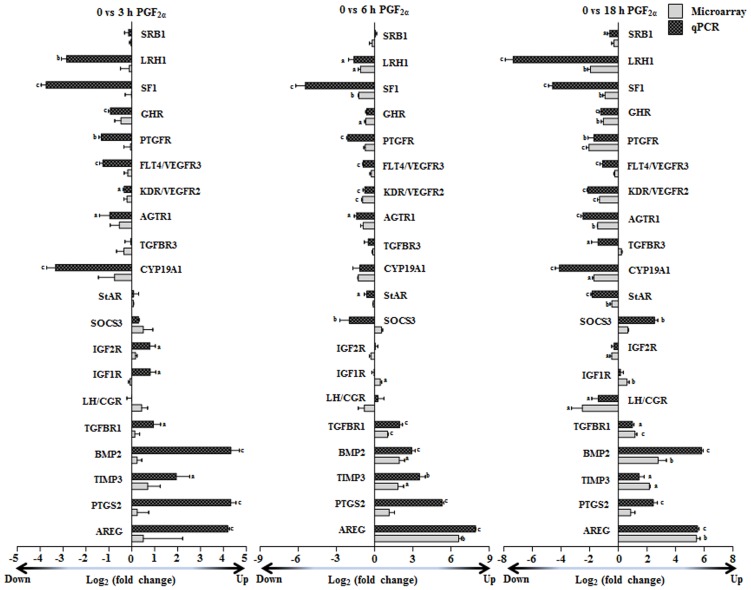
Validation of microarray data and its comparison with qPCR analysis. Log ratio of microarray fold change expression of the selected 20 up and down regulated genes associated with specific biological process at 3, 6 and 18 h post PGF_2α_ administration in the CL. The genes selected by microarray analysis were subjected to qPCR analysis and log ratio of fold expression changes at different time points post PGF_2α_ administration are represented as bar graphs. Individual bar for each gene represents mean±SEM log_2_ (fold change) in mRNA expression value for microarray analysis and qPCR analysis at each time point (n = 3 animals/time point). For each gene, bars with different alphabets above them are significantly different (p<0.05).

Figure 2 shows fold changes in mRNA expression of 20 differentially expressed genes of both microarray and qPCR analyses. Of the 20 differentially genes analyzed by qPCR analysis, the results of 15 genes (CYP19A1, TGFBR3, AGTR1, VEGFR2/KDR, VEGFR3/FLT4, AREG, PTGS2, TIMP3, BMP2, TGFBR1, PTGFR/FPR, GHR, IGF1R, IGF2R and StAR) were also utilized for validation of microarray data (i.e. for validation and correlation between microarray and qPCR analyses was performed and the results presented in Figure 1C). The goodness of fit analysis showed that the fold change in qPCR analysis of mRNA expression data corroborated well with the microarray analysis data (Figure 1C). At 3 h post PGF_2α_ administration the correlation coefficient ‘r’ value was 0.69 (p<0.003, Figure 1C), while the ‘r’ value at both 6 and 18 h time points was 0.89 (p<0.0001, Figure 1C). Many of the differentially expressed genes were selected for qPCR analysis to further characterize their expression in relation to their function such as signaling pathways (for pathway activity analysis), key enzymes/proteins associated with the steroidogenesis, growth factor activation status, prostaglandin biosynthesis, activation of cytokine signaling and genes associated with extracellular matrix (Figure 2). Expression analysis data of select genes presented in Figure 2 also provide information as to the time course changes in expression post PGF_2α_ treatment. Comparison of expression data of select genes between microarray and qPCR analyses at 3 h time point revealed that the fold expression changes by microarray analysis was lower compared to other time points, but fold change in expression of same genes by qPCR analysis was higher (Figure 2). Similar comparison analyses at 6 and 18 h time point revealed similar observation, except for the gene expression of SRB1 (at 6 h) and LHCGR, IGF1R, IGF2R, PTGFR (at 18 h) which showed higher fold change expression changes by microarray than qPCR analysis.

The top fifteen differentially expressed genes at the three time points (3, 6 and 18 h) post PGF_2α_ administration were determined and the data are represented as list of top most up- and down-regulated genes in a tabular format comprising of probe set ID, fold change compared to untreated control time point (0 h), gene ID and gene title [from Entrez Gene or UniGene] (Tables S2, S3, S4, S5, S6, S7).

### Pathway analysis of differentially expressed genes employing Ingenuity pathway analysis (IPA) software

**Figure 3:**
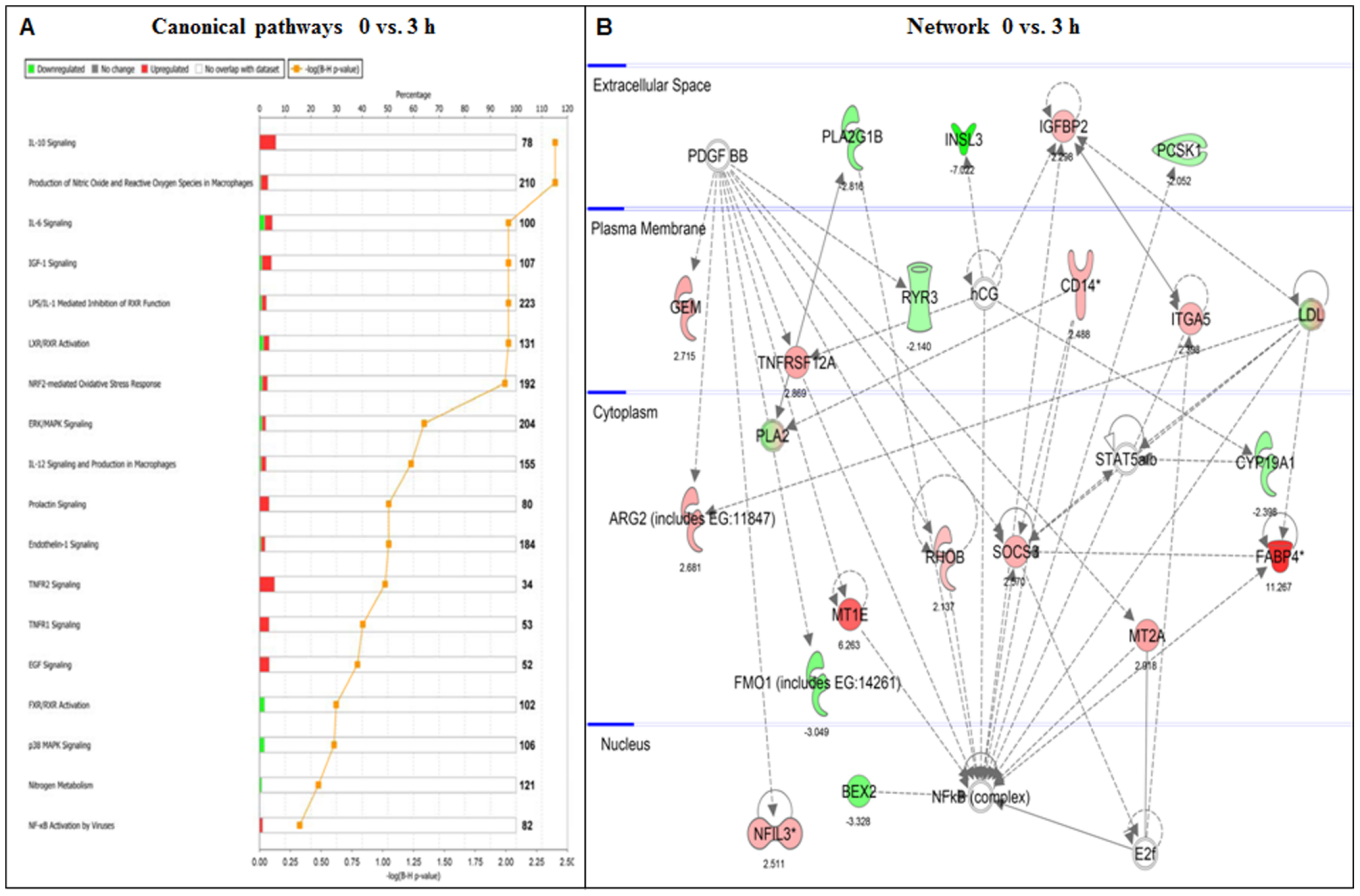
Ingenuity Pathway Analysis (IPA) of differentially expressed genes. (A) The pathway analysis indicated that a large number of differentially expressed genes belong to canonical pathways such as IGF-1 signaling, steroidogenesis, chemokine signaling, prolactin signaling, cellular growth and proliferation, extracellular matrix modulation and apoptosis. The orange line represents score for the likelihood [-log (B-H p<0.05)] that genes belonging to a specific canonical pathway category affected at 3 h post PGF_2α_ administration. The stacked bars indicate the percentage of genes distributed according to regulation, i.e., green (down), red (up) and open bars (no overlap with dataset) in each canonical pathway. (B) Network 0 vs. 3 h: Ingenuity Pathway Analysis of the differentially regulated genes 3 h post PGF_2α_ administration shows a network of 21 focus molecules with a score of 48, with top biological functions of cell death, cellular growth and proliferation (immune cells), and cell-to-cell signaling. The network is displayed graphically as nodes (genes/gene products) and edges (biological relationship between nodes). The node color intensity indicates the fold change expression of genes; with red representing up-regulation, and green representing down-regulation of genes between 0 vs. 3 h post PGF_2α_ administration. The fold change value for individual gene is indicated under each node. The shapes of nodes indicate the functional class of the gene product and the lines indicate the type of interaction.

The differentially expressed genes identified in CL tissues post PGF_2α_ treatment were classified into different functions. The IPA analysis revealed that 85–90% of the differentially expressed genes were observed to be function or pathway eligible. Gene classification according to the canonical signaling pathways indicated that many of the differentially expressed genes were associated with process and/or function of IGF-1 signaling, steroidogenesis, chemokine signaling, prolactin signaling, cellular growth and proliferation, extracellular matrix modulation and apoptosis. Further, performing IPA on the differentially expressed genes, 42 networks were identified, out of which 16 networks had 20 or more focus genes in each network. Figure 3A&B shows different canonical signaling pathways and genes identified in Network for 0 vs. 3 h time point (score 48, 21 focus molecules, Figure 3B). The network of genes are associated with growth factor signaling, cytokine signaling, steroidogenesis and extracellular matrix The classification of canonical pathways and network of genes for 6 and 18 h time points post PGF_2α_ treatment are provided in Figure S3 and S4, respectively.

### Effect of PGF_2α_ administration on expression of orphan nuclear transcription factors associated with regulation of steroidogenesis in the CL

Since the effects of PGF_2α_ on PKC activation pathway are well recognized, its effects on steroidogenesis at the molecular and biochemical levels were examined. Both microarray and qPCR analyses of mRNA expression of orphan nuclear transcription factors NR5A1/SF-1 and NR5A2/LRH-1 involved in the regulation of expression of steroidogenic genes showed down regulation, excepting at 3 h time point for SF-1, at all-time points post PGF_2α_ treatment (Figure 2). Immunoblot analyses for protein of both these genes also showed down regulation similar to their mRNA expression patterns (Figure S5A&B).

### Effect of PGF_2α_ treatment on LH/CGR activation

**Figure 4:**
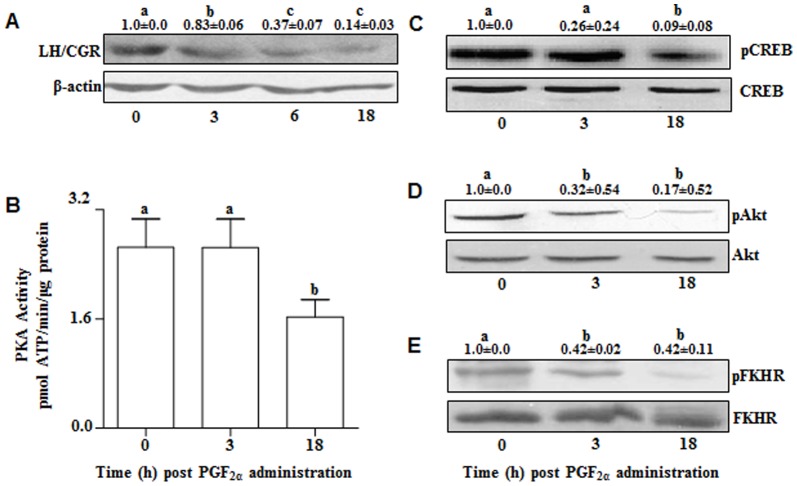
Effects of PGF_2α_ on expression of LH/CGR and downstream signaling molecules, Akt and FKHR. (A) Protein levels of LH/CGR in bovine CL. Protein lysate (100 µg) prepared from CL tissue collected before (0 h) and post (3, 6 and 18 h) PGF_2α_ treatment were resolved on 10% SDS PAGE, transferred onto PVDF membrane and immunoblot analysis was performed using anti-LHCGR and anti-β-actin antibody. A representative immunoblot for each of the antibody probed is shown. The immunoblot probed with β-actin antibody indicates loading control for each lane. Densitometric values were determined and indicated as mean±SEM (n = 3 animals/time point), relative to intensity of β-actin for each time point post PGF_2α_ treatment. The values of immunoblot analysis has been put on top of each lane and lane with different alphabets indicate statistical significance, p<0.05. (B) PKA activity in the bovine CL before (0 h) and after (3 and 18 h) PGF_2α_ administration. Values represent mean±SEM for each time point post PGF_2α_ administration (n = 3 animals/time point). Individual bars with different alphabets indicate statistical significance (p<0.05). (C, D and E) Protein levels of pCREB, CREB, pAkt, Akt, pFKHR and FKHR in the CL. Protein lysate (50 µg) of each CL tissue collected at different time points was subjected to immunoblot analysis employing anti-pCREB, anti-CREB, anti-pAkt, anti-Akt, anti-pFKHR, anti-FKHR antibodies. A representative immunoblot for each of the antibody probed is shown. Densitometric values were determined and represented as mean±SEM (n = 3 animals/time point), relative to intensity of total CREB (C), Akt (D) and FKHR (E) for each time point post PGF_2α_ treatment. The values of immunoblot analysis has been put on top of each lane and lanes with different alphabets indicate statistical significance (p<0.05).

Although LH/CGR mRNA expression by microarray and qPCR analyses indicated marginal decrease at 6 and 18 h post PGF_2α_ treatment (Figure 2), but the protein expression was significantly (p<0.05) lower post PGF_2α_ treatment (Figure 4A). To further examine whether PGF_2α_ treatment affected signaling molecules downstream of LH/CGR activation, PKA activity and CREB phosphorylation levels were determined and the results indicated decreased PKA activity (Figure 4B) and lowered pCREB levels (Figure 4C) at 3 and 18 h time points examined. Also, a decreased phosphorylated Akt (Figure 4D) and FKHR (Figure 4E) levels post PGF_2α_ treatment suggesting inhibitory effects on survival signaling pathway.

### Effect of PGF_2α_ treatment on circulating and luteal E_2_ levels and expression of E_2_ responsive genes in the CL

**Figure 5:**
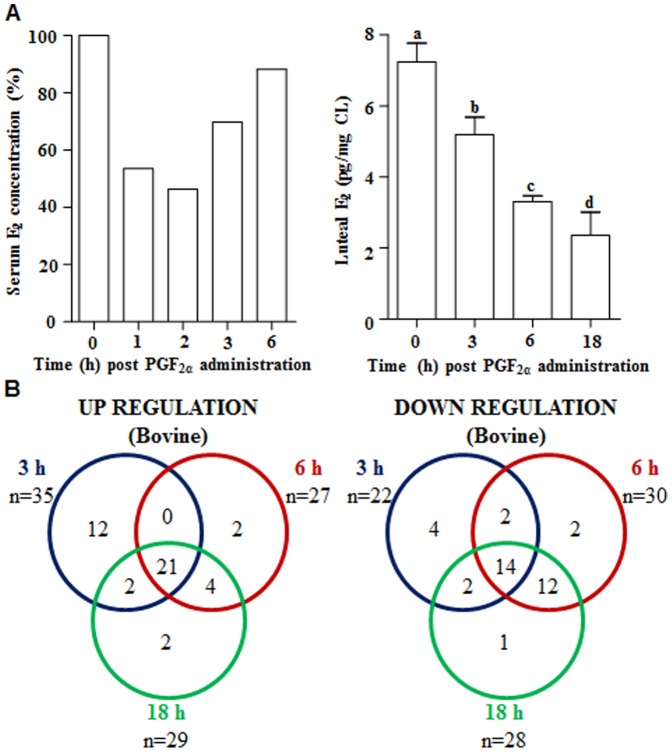
Effects of PGF_2α_ treatment on circulating and luteal E_2_ levels and E_2_ responsive genes. (A) Circulating serum and luteal estradiol (E_2_) concentration at different time points post PGF_2α_ treatment. Values from animals (n = 3/time point) were represented as mean±SEM and bars with different alphabets indicate statistical significance, p<0.05. (B) Venn diagrams representing the number of differentially expressed E_2_ responsive genes identified after microarray analysis of CL tissues collected at different time points post PGF_2α_ treatment. Identification of potential E_2_ responsive genes from the differentially expressed genes. Identification of genes as target of E_2_ action was based on PCR array human estrogen signaling, estrogen responsive gene database and classical E_2_ responsive genes. Data analyzed by Bioconductor analysis tool employing ≥1 fold change cut-off and statistical filters with Benjamini and Hochberg correction factor for false discovery rate. The comparison of total number of differentially expressed up and down regulated E_2_ responsive genes found common between 0 vs. 3 h (blue circle) and 0 vs. 6 h (red circle), as well as between 0 vs.18 h (green circle) post PGF_2α_ administration are presented.

Of the many steroidogenic genes which were affected by PGF_2α_ treatment, mRNA expression of CYP19A1 gene that codes for aromatase enzyme responsible for conversion of androgens to estrogens was down regulated (see Figure 2) at all the time points examined. Figure 5A shows circulating and luteal E_2_ levels before and after PGF_2α_ treatment. Due to variations in circulating E_2_ concentrations between animals, the pre-treatment concentration of E_2_ in each animal was set as 100% and values post PGF_2α_ treatment were expressed in relation to 100%. As can be seen from Figure 5A, E_2_ concentrations decreased significantly within 1 h after PGF_2α_ treatment and continued to be lower until 6 h. Since % decrease in E_2_ was not seen after 6 h post treatment this was perhaps due to contribution of E_2_ from increased growth and development of ovarian follicles. To further validate the effects of PGF_2α_ on E_2_ synthesis and secretion, luteal E_2_ levels were monitored and the results are presented in Figure 5A. As can be seen from the figure, luteal E_2_ levels continued to decline post PGF_2α_ treatment.

Luteal expression of E_2_ receptors, α and β, during the luteal phase of estrous cycle and their expression patterns post PGF_2α_ treatment are presented in Figure S6. Since estrogen has both local and systemic effects, expression of estrogen responsive genes in the CL tissue was examined. For examining the effects of PGF_2α_ treatment on expression of E_2_ responsive genes in the CL tissue, in addition to the classical E_2_ responsive genes [54], genes identified as target of E_2_ action by PCR array human estrogen signaling (Qiagen, SABiosciences, PHAS-005A) as well as contents of estrogen responsive gene data base (ERGDB) were included. In all, a total of 89 genes identified as potential E_2_ responsive genes were considered for analysis. From the list of differentially expressed genes obtained by microarray analysis in CL tissue at different time points post PGF_2α_ treatment, as many as 57 out of 89 E_2_ responsive genes (both up and down regulated) were identified to be differentially expressed at least 1 fold or more. The data is represented as Venn diagram in Figure 5B. The list of E_2_ responsive genes for 3 h time point is shown in Tables S8, S9. The list for other time points is not shown since expression of large number of E_2_ responsive genes were commonly regulated at all-time points. The list of E_2_ responsive genes commonly up and down regulated at all-time points post PGF_2α_ is shown in Tables S10, S11. Twenty one of the 43 up regulated genes were observed to be commonly regulated at all-time points examined, while 37 genes identified as down regulated, 14 were found to be commonly regulated at all-time points post PGF_2α_ treatment (Figure 5B).

**Figure 6:**
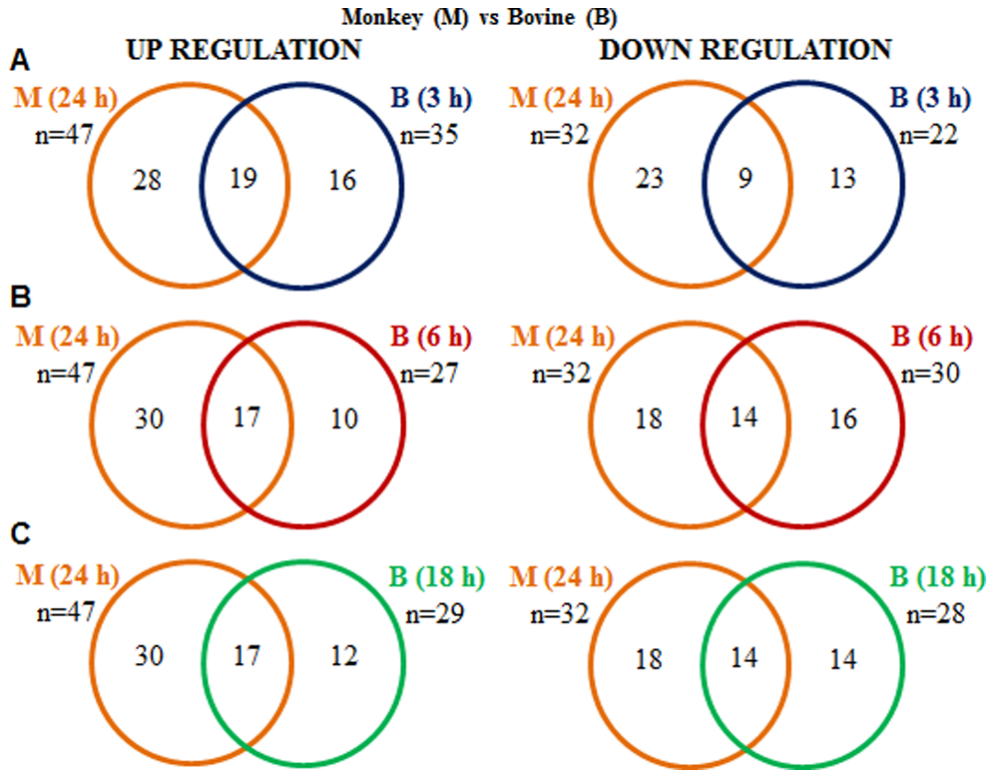
Identification and comparison of differentially expressed E_2_ responsive genes in the monkey and bovine CL. Venn diagrams representing the number of differentially expressed E_2_ responsive genes identified in monkey (M) and bovine (B) microarray data available from the GEO database. Data analyzed by Bioconductor analysis tool employing ≥1 fold change cut-off and statistical filters with Benjamini and Hochberg correction factor for false discovery rate. The circles designated for each time point post PGF_2α_ is represented as orange (24 h, M), blue (3 h, B), red (6 h, B) and green (18 h, B). A comparison of total number of differentially expressed up and down regulated E_2_ responsive genes found common between 24 h, M vs. 3 h, B (A); 24 h, M vs. 6 h, B (B) and 24 h, M vs. 18 h, B (C) post PGF_2α_ treatment are presented.

The buffalo CL much like the cattle is not regarded as a site of high E_2_ production since much of the circulating E_2_ in bovines is contributed by waves of follicular growth occurring throughout the estrous cycle [55], [56]. On the other hand, in species such as primates, CL is the predominant source of E_2_ production throughout the luteal phase period and circulating E_2_ contributed by the functional CL is considerable [57]–[59]. To further validate our hypothesis that decreased estrogen production following inhibition of CYP19A1 expression might adversely affect CL function and its eventual survival, the previously published microarray data of the differentially expressed genes from the CL tissues of macaques receiving PGF_2α_ treatment for 24 h ([44], GEO accession number GSE8371) was mined for E_2_ responsive genes (89 genes identified as potential E_2_ responsive genes, see above) for purposes of comparing the number of E_2_ responsive genes that were differentially expressed in CL of macaques (in which E_2_ is secreted in higher amounts) to that of the buffalo cow CL (in which E_2_ secretion is low). The mined data comprising up and down regulated genes of CL from macaques at 24 h time point (the single time point for which microarray analysis available) vs. E_2_ target genes identified at 3, 6 and 18 h time points of buffalo CL tissue post PGF_2α_ treatment are shown in Figure 6 (A–C). Forty seven out of the 89 genes regarded as E_2_ responsive genes were identified as up regulated in the monkey CL, 24 h post PGF_2α_ treatment and 19 of the 47 genes were also observed to be commonly regulated in CL tissues of buffalo cows post 3 h PGF_2α_ treatment (Figure 6A). Remarkably, the number of down regulated genes identified in CL tissues at 24 h post PGF_2α_ treatment in macaques and at 6 and 18 h post PGF_2α_ treatment in buffalo cows were similar and 14 of the genes were found to be commonly regulated between the two species (Figure 6B&C). The list of E_2_ responsive genes commonly up and down regulated from macaques at 24 h time point vs. genes identified at 3, 6 and 18 h time points of buffalo CL tissue post PGF_2α_ treatment is shown in Tables S12, S13, S14, S15, S16, S17.

### Effect of PGF_2α_ administration on expression of different regulatory molecules associated with cell survival and apoptosis

**Figure 7:**
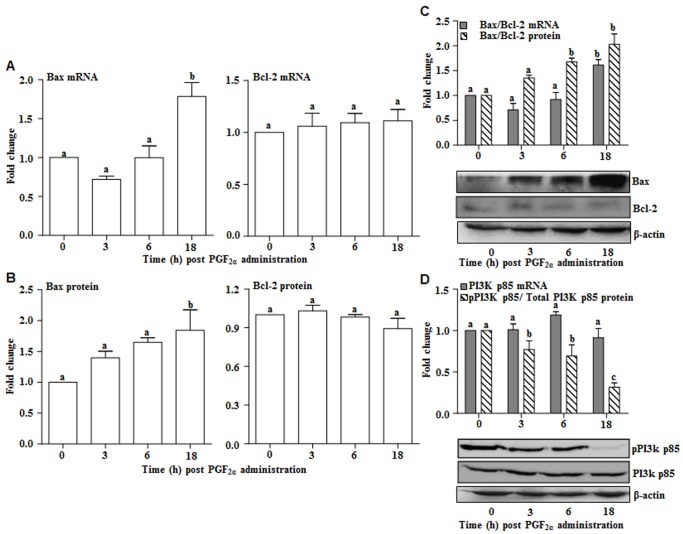
Effect of PGF2α on expression of Bax, Bcl-2 and Akt genes during PGF2α treatment. (A) qPCR expression and analysis of Bax and Bcl-2 in the bovine CL. Mean (±SEM) fold expression changes before and after PGF_2α_ treatment is presented. Bars with different alphabets above them indicate statistical `significance (p<0.05). (B) Protein levels of Bax and Bcl-2 in the bovine CL. The CL tissue lysates were resolved on 10% SDS PAGE, transferred onto PVDF membrane and subjected to immunoblot analysis employing anti-Bax, anti-Bcl-2 and anti-β-actin antibodies. The immunoblot probe with β-actin antibody was used as a loading control. Densitometric values were determined and indicated as mean±SEM (n = 3 animals/time point), relative to intensity of β-actin for each time point post PGF_2α_ treatment. Bars with different alphabets indicate statistical significance (p<0.05). (C) Fold expression changes in mRNA and quantitative changes in protein as ratio of Bax/Bcl-2 levels in the luteal tissue during PGF_2α_ treatment. A representative immunoblot for each of the antibody probe is shown. Individual bars with different alphabets above them indicate statistical significance (p<0.05). Individual bars in solid box and open box represent mRNA expression and protein levels, respectively. (D) mRNA and protein levels of PI3k p85 in bovine CL. Mean (±SEM) fold expression changes in PI3k p85 mRNA examined by qPCR analysis. Quantitative changes in the ratio of pPI3k p85 and PI3k p85 protein levels were estimated. For the protein loading control, blots were probed with β-actin antibody. Densitometric values were determined and represented as mean±SEM (n =  3 animals/time point), relative to intensity of total PI3k p85 for each time point post PGF_2α_ treatment. Individual bars with different alphabets above them indicate statistical significance (p<0.05). Individual bars in solid box and open box represent mRNA expression and protein levels, respectively.

To examine whether down regulation of E_2_ receptor expression results in activation of apoptotic changes in the luteal tissue, expression of Bax and Bcl-2 genes were determined in the luteal tissue post PGF_2α_ treatment. Figure 7A&B illustrates expression of (both mRNA and protein) Bcl-2 family members, Bax (pro-apoptotic) and Bcl-2 (anti-apoptotic) during PGF_2α_-induced luteolysis. The Bcl-2 mRNA expression was unchanged post PGF_2α_ treatment, but a significant increase in Bax mRNA expression was seen at 18 h post PGF_2α_ treatment (Figure 7A). Similarly, at the protein levels, Bcl-2 protein levels did not change, but a significant increase was seen for Bax protein at 18 h time point post PGF_2α_ treatment (Figure 7B). As indices of activation or inhibition of mitochondrial permeability to apoptogenic molecules, changes in Bax and Bcl-2 mRNA expression and protein levels were expressed as Bax/Bcl-2 ratios. A significant increase in both Bax mRNA and protein levels post PGF_2α_ treatment was observed (Figure 7A&B). As can be seen from Figure 7C, Bax/Bcl-2 ratio for both mRNA and protein levels, increased significantly by 18 h post PGF_2α_ treatment. Also, a significant increase in Bax/Bcl-2 protein was observed by 6 h post PGF_2α_ treatment suggesting increased mitochondrial permeability to apoptogenic molecules (Figure 7C).

It has been reported that PI3K activity is essential for the activation of Akt for E_2_ actions [60], [61]. In present study, although a significant change in the expression of PI3K p85 mRNA by qPCR analysis was not observed (Figure 7D), but a significant decrease in phospho levels of PI3K p85 was observed at all-time points post PGF_2α_ treatment (Figure 7D).

### Schematic diagram illustrating a model for interaction amongst different signaling pathways during PGF_2α_-induced luteolysis in buffalo cows

**Figure 8:**
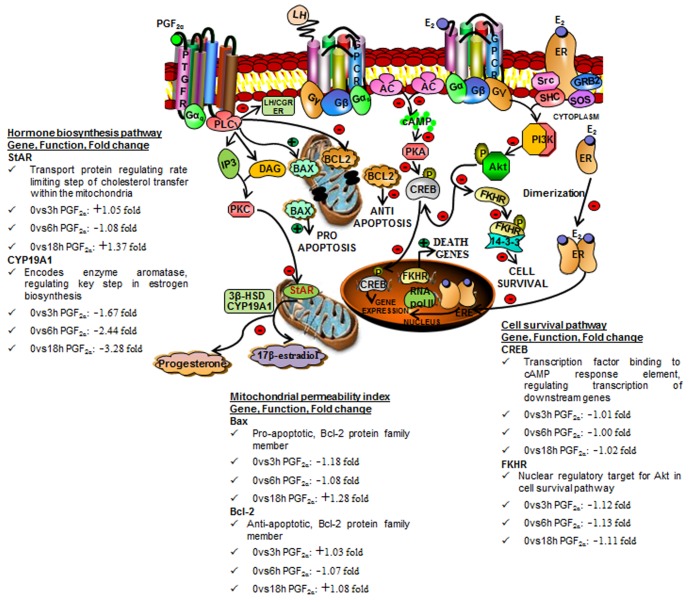
Schematic diagram illustrating a model for PGF_2α_-induced luteolysis in the bovine corpus luteum. The model shows interaction amongst various intracellular signaling pathways activated by LH, PGF_2α_ and E_2_. The luteolysis process induced by PGF_2α_ treatment appears to be a result of distinct molecules that involve hormone biosynthesis pathways (StAR, CYP19A1) which is downstream of PTGFR signaling leading to decrease in serum and luteal P_4_ and E_2_ levels. The model also shows involvement of Bcl-2 family members, Bax (pro-apoptotic) and Bcl-2 (anti-apoptotic) that cause changes in mitochondrial permeability to apoptogenic molecules. The model also shows inhibition of cell survival pathway as shown by LH/CGR and ER (both genomic and non-genomic signaling) down regulation and in turn inhibition of downstream molecules (CREB, FKHR). Few of the selected genes out of differentially expressed genes at different time points post PGF_2α_ treatment compared with 0 h PGF_2α_ treatment (StAR, CYP19A1, Bax, Bcl-2, CREB, FKHR) has been provided in the Figure. The information on gene names, general function, fold change in mRNA expression and implicated pathways are represented. The stimulation and inhibition of specific molecules downstream of different signaling pathways are represented by green (+) and red (−) signs, respectively.

Extensive studies carried out previously by several groups have identified different molecules of the signaling pathways for LH and PGF_2α_ actions in the luteal tissue of various species [62]–[66]. In the present study, some of the signaling molecules of both these pathways were analysed as also genes associated with steroid biosynthesis, cell survival and apoptosis and the data for expression of various genes and their protein levels in the luteal tissue at different time points post PGF_2α_ treatment are presented in Figure 8. A schematic diagram of LH and PGF_2α_ signaling pathways and E_2_ receptor signaling pathways and their likely interactions is depicted in Figure 8. In the proposed model, an association between cell survival and E_2_ action could be suggested for regulating luteal function in the bovine luteal tissue. The down regulation of E_2_ signaling by PGF_2α_ as suggested by the data indicates activation of apoptotic pathway (Figure 8).

## Discussion

In bovines, during non-conception reproductive cycles, the onset of spontaneous luteolysis is initiated by increased pulsatile secretion of PGF_2α_ from the uterus [62], [67]. Several recent studies have compared expression changes during periods of refractoriness and responsiveness of CL to PGF_2α_ treatment [23], [28], [68] and few studies have previously reported global gene expression changes during PGF_2α_ induced luteolysis [23], [26]. In the present study, employing high throughput screening technique, genome-wide gene expression changes in the CL of buffalo cow during PGF_2α_-induced luteolysis were determined. The primary objective of this study was to examine temporal expression changes during early periods of PGF_2α_ treatment in which functional loss in CL will be manifest. The global transcriptome changes post PGF_2α_ treatment revealed temporal increases in the expression of a number of differentially expressed genes at 3, 6 and 18 h time points examined, and of these several of them were immediate early genes. The analysis of differentially expressed genes during induced luteolysis also revealed cluster of genes associated with steroidogenesis, angiogenesis, cell survival and apoptosis and these have major bearing on initiation of functional luteolysis and apoptotic processes. Further, comparison of differentially expressed genes data from the present study with other studies following administration of PGF_2α_ treatment in bovine and primate species indicates gene expression changes related to angiogenesis, immune system and participation of functional changes in non-steroidogenic cells [44], [23].

Analysis of differentially expressed genes at 3 h post PGF_2α_ treatment indicated that many of the genes were associated with canonical cell to cell signaling pathways (chemokine, IGF-I and prolactin signaling), cellular growth and proliferation. Several of the products of the up-regulated genes (TNFRSF12A, DAPL1, GEM, PPP4R4 and OSR2) were associated with activation of cellular signaling resulting in inhibition of aggregation of denatured proteins as well as processes associated with protection against metal toxicity and oxidative stress (FABP4, HSPH1, MT1A, MT2A, HP and CBR1). Many of the down-regulated genes belonged to steroidogenesis process (CYP19A1, NR5A1/SF-1, NR5A2/LRH-1, PLA2G1B and PTGR1), cell cycle regulation and tissue development (INSL3, AHSG, BEX2, CDKN1C and HSPB3). Earlier studies involving intra-uterine infusions of low [23] and pulsatile [3] administration of PGF_2α_ also observed similar changes in many of the genes during functional luteolysis. It should be pointed out that expression of genes regulating steroidogenesis i.e. P_4_ biosynthesis (LH/CGR, P450scc and HMGCR) or expression of genes involved in the uptake and trafficking of cholesterol (SR-B1 and StAR) did not change significantly at 3 h post PGF_2α_ treatment. However, changes in expression of many of these genes were observed at later time points. It has been reported that P_4_ decline occurs as early as 30 min after PGF_2α_ treatment by others and us [69], [70] suggest translational or post translational changes are responsible for initiation of rapid induction of functional luteolysis.

To examine the mechanism of PGF_2α_-induced luteolysis, the differentially expressed genes belonging to some processes and functions of luteal cells and few top early differentially expressed genes were subjected to additional analysis. More specifically, as per the objectives of the present study stated in the introduction section, effects of PGF_2α_ on luteotrophic signaling and role of intra luteal factors were further examined from the differentially expressed genes post PGF_2α_ treatment. Several studies have reported inhibition of LH-stimulated P_4_ secretion by PGF_2α_ [71], [72]. One of the reported actions of PGF_2α_ was inhibition of adenylate cyclase activity [73], [74] that lie downstream of LH receptor activation, but this effect has been reported to be a transient one and moreover, this effect appears to be limited to small luteal cells [75], [76]. It is not clear to what extent PGF_2α_-inhibited adenylate cyclase activity will adversely affect overall P_4_ production *in vivo*, since large luteal cells, besides having large volume and being most abundant, have constitutive steroid synthesis and therefore high P_4_ production capacity [63]. On the other hand, the LH-responsive small luteal cells contribute very small fraction of total P_4_ produced from the luteal tissue. It should be pointed out that fewer pulses of LH are secreted during the luteal phase that may have little or no impact on the overall P_4_ production capacity of the CL [77]–[80]. It has been reported that LH/CGR expression gets inhibited post PGF_2α_ administration in several species [71], [72]. In the present study, LH/CGR mRNA expression did not change within 3 h, but decreased at 6 h and thereafter. Surprisingly, LH/CGR protein levels decreased within 3 h post PGF_2α_ treatment and this may be the reason for immediate inhibitory effect observed in luteal cells as reported in bovines [81] and other species [11], [72]. The decrease in PKA activity and pCREB levels at later time points post PGF_2α_ treatment suggest that aspects of LH/CGR signaling appears to be modulated by PGF_2α_ directly at the cellular level through inhibition of LH/CGR protein or cAMP levels due to inhibition of cAMP phosphodiesterase enzyme [82], [83]. A decrease in levels of SF1 and LRH1 associated with regulation of steroidogenesis indicates regulation of expression of early genes involved in modulation of steroidogenesis [84].

One of the novel findings of this study is the early and consistent down regulation of CYP19A1 expression post PGF_2α_ treatment. It has been established that in response to LH surge, CYP19A1 expression gets down regulated and may even be absent post ovulation [85]–[87]. However, others [36] and we (in the present study) have observed CYP19A1 expression in the non-pregnant CL. It was of interest to examine the importance of PGF_2α_-induced down regulation of CYP19A1 expression. The aromatase enzyme, encoded by CYP19A1 gene, a complex composed of two proteins, an ubiquitous NADPH cytochrome P450 reductase and the cytochrome P450 aromatase, is essential for conversion of androstenedione to E_2_. It has been determined that the regulatory sequence, cAMP-response element (CRE) on the promoter region of CYP19A1 gene is critical for control of expression of aromatase in mammals [88]. For driving expression of CYP19 gene, many tissue specific promoters have been described for several species and in the ovary, promoter II sequence has been reported to be responsible for driving aromatase expression [89]–[91]. Although the promoter II sequence in several species including the cow presents strong homologies, differences in the regulatory sequence have been observed amongst species [92], [93]. In the cow, one nucleotide deletion and two substitutions in the CRE-like sequence (CLS) leads to inactivation of the promoter II site and this has been suggested to be responsible for the presence of very low aromatase activity in luteinized granulosa cells or in the CL tissue [92], [94]. Interestingly, CYP19 gene expression is most abundant in CL of pregnant rats [92], and this has important role since large quantities of androstenedione, the substrate for the aromatase enzyme, is synthesized in the placenta but gets converted to E_2_ in the luteal tissue [95], [96]. In bovines, even though CYP19 expression may be lower in non-pregnant CL, but it may be higher during pregnancy [97].

Intra luteal E_2_ plays an important role in the structure and function of CL ranging from hypertrophy of luteal cells to increased transport of cholesterol for steroidogenesis [98]–[100]. Estrogens have been reported to have both luteotrophic and luteolytic function across different species [15], [36], [38], [39], [64], [101], which suggest autocrine/paracrine role within the CL. The biological effects of E_2_ are mediated by its binding to the structurally and functionally distinct estrogen receptor (ER), α and β. It has been documented that besides classical E_2_ signaling, ligand activated ERα regulates a series of non-genomic events in the cytoplasm including PI3-kinase (PI3K) activation [61], [102], [103]. The present observation of decreased ER expression and inhibition of Akt phosphorylation post PGF_2α_ treatment point to loss of PI3K activity during luteal regression as reported previously by others [25], [37]. Further, in order to gain more information on the effects of E_2_ on the CL tissue, the microarray data of PGF_2α_ treatment study was mined for E_2_-responsive genes. The observation that a large number of E_2_-responsive genes was observed to be differentially expressed post PGF_2α_ treatment was surprising since intra luteal E_2_ levels are considerably lower in the bovine CL tissue (and also in the circulation) and therefore, it was reasoned that influence of E_2_ on luteal function, if any, will be limited. However, to further explore the possible E_2_ effects on luteal tissue, effects of PGF_2α_ treatment on expression of genes in CL tissues of macaques, the species considered to secrete large quantities of E_2_ [104] were determined. Mining of microarray data for E_2_-responsive genes from CL tissues of macaques receiving PGF_2α_ treatment [44] revealed that many of the E_2_ responsive genes were also observed to be differentially expressed post PGF_2α_ treatment in the macaque CL. The observation of many of the differentially expressed E_2_-responsive genes in CL of both macaque and bovine species post PGF_2α_ treatment provides compelling evidence for regulation of CL function by E_2_. Furthermore, PGF_2α_ appears to interfere with luteal E_2_ secretion and perhaps E_2_ actions.

Earlier studies by others and us have provided evidence supporting involvement of cohort of conserved cell death regulatory factors, required for maintenance of growth and development of CL across species [8], [9], [105], [106], [107]. Apoptosis at cellular levels is regulated by the intricate changes in the members of the Bcl-2 family proteins that are classified either as anti-apoptotic (Bcl-2 and Bcl-xL) or pro-apoptotic (Bax, Bad, Bik, Bid) molecules. Bcl-2 has been designated as estrogen responsive gene [108], [109] and up regulation of Bcl-2 expression has been identified as a critical mechanism for promoting cell survival. Akt mediated phosphorylation of cytosolic proteins is known to play a critical role in the regulation of different metabolic pathways. The promoter region of Bcl-2 contains a cAMP responsive element (CRE) site which is flanked by two estrogen response element (ERE) sites [103], [110], [111], [112]. Based on the previous studies, the transcription factor, CREB can be considered as a positive regulator of Bcl-2 expression [111], [113]. Further, Akt, a target of E_2_ signaling through PI3K pathway has been shown to activate CREB [103], [114] thereby regulating Bcl-2 expression via differential activation of ERs. In our present study, an increase in the Bax/Bcl-2 ratio at mRNA and protein levels further suggests an important role played by permeability of these regulatory proteins across mitochondria as reported during induced and spontaneous luteolysis in bovines [8], [9], [63]. Another regulatory molecule FKHR which is target of Akt has been proposed to participate in both metabolic and cell survival pathway [115], [116], has also been observed to decline post PGF_2α_ treatment, activating the apoptotic pathway.

The other effects of PGF_2α_ involve interaction with intraovarian paracrine and endocrine factors [117], [118] to mediate intracellular communication within the CL [119]. PGF_2α_-induced structural luteolysis depends on cell composition (immune or endothelial cell) [120] and contact [121] as the vasoconstrictor property of PGF_2α_ may decrease ovarian blood flow and cause apoptosis of endothelial cells [65], [122]–[124] restricting access of gonadotropins and oxygen to steroidogenic cells. The possible role of apoptosis as a mechanism for structural luteolysis has received considerable attention in several species [125], [126]. The PGF_2α_-induced structural luteolysis mediated by apoptosis was confirmed by the presence of DNA laddering in 18 h PGF_2α_-treated CL, which has been demonstrated by us and others through studies involving the role of Bax, Bcl-2, Fas/FasL and caspases during spontaneous or PGF_2α_-induced apoptosis in ovine, bovine, rodent CL [9], [107], [127]. Thus, pathway analysis of microarray data at 6 and 18 h post PGF_2α_ administration using IPA revealed down-regulated genes to be associated with regulation of steroidogenesis (transcription factors belonging to steroidogenesis, biosynthesis of steroids, intracellular trafficking and lipid metabolism) and angiogenesis (VEGF signaling and coagulation system). Whereas, up-regulated genes to be associated with cell-cell interaction and signaling (EGF, p38 MAP kinase, NF-κB, TGF-β and apoptotic signaling), prostaglandin metabolism and tissue remodelling. Analysis of top differentially regulated genes indicated that most genes belonged to steroidogenesis, prostaglandin metabolism, tissue remodeling or ECM modulation, angiogenesis, TGF-β signaling, cellular stress activated binding proteins, intracellular trafficking, cell survival and apoptotic signaling.

In summary, the results of the present study describe gene expression changes at early time points post PGF_2α_ treatment. It was observed that PGF_2α_ treatment caused down regulation of various components of LH receptor signaling, decreased CYP19A1 expression and inhibited intra luteal E_2_ levels. A number of E_2_ responsive genes were identified to be differentially expressed post PGF_2α_ treatment. In conclusion, based on the changes of key genes encoding proteins involved in regulating CL structure and function, we propose a model depicting a cross talk between PGF_2α_, LH/CGR and E_2_ signaling during luteolysis in bovines. PGF_2α_-induced luteolysis involves down and up regulation of genes involved in luteal steroidognesis and susceptibility of cell system to apoptotic signals, respectively. The distinct changes that follow post PGF_2α_ treatment is triggered by down regulation of LH/CGR and ER signaling. E_2_-ER/PI3K-Akt signaling regulates many transcriptional regulatory molecules such as CREB and FKHR, and members of Bcl-2 family proteins such as Bax and Bcl-2. Thus, the present study suggests key role of inhibition of E_2_ signaling in the regulation of various changes observed during PGF_2α_-induced luteolysis in bovines.

## Supporting Information

Figure S1 **Expanded tree view generated after hierarchical clustering of representative genes obtained after pairwise analysis.** The expanded tree displaying the hierarchy analysis for probe sets at each time point [0 vs. 3 h (A), 0 vs. 6 h (B) and 0 vs. 18 h (C)] post PGF_2α_ administration. The bottom of each dendrogram shows the condition color bar with the parameters in each interpretation. The legend shows the name of each condition on which clustering was performed. Header of heat map shows a normalized intensity values represented in various shades of red and green indicating relatively either up or down regulation, respectively. The row header shown on the right side represents the complete entity name or gene symbol. The upper panel shows groups of genes, whose expression was up regulated at 0 h and with the administration of PGF_2α_ the expression is observed to be down-regulated. The lower panel shows groups of genes whose expression was down regulated at 0 h and with the administration of PGF_2α_ the expression is observed to be up regulated.
(TIF)

Figure S2 **Effects of PGF_2α_ on circulating and luteal P_4_ levels, StAR expression and DNA fragmentation.** Buffalo cows received intramuscular injection of 500 µg of PGF_2α_ on day 11 of estrous cycle and blood and luteal tissue samples at different time intervals post PGF_2α_ treatment. (A) Circulating mean±SEM serum and luteal progesterone (P_4_) concentrations immediately before (0 h) and at different time points post PGF_2α_ treatment. Bars with different alphabets indicate statistical significance, p<0.05. (B) Protein lysate (100 µg) prepared from CL tissue collected before and post PGF_2α_ treatment were resolved on 10% SDS PAGE, transferred onto PVDF membrane and immunoblot analysis was performed using anti-StAR and anti-β-actin antibody (β-actin was used as loading control). A representative immunoblot for each of the antibody probed is shown. Densitometric values shown on top of each lane represented were determined and represented as mean±SEM (n = 3 CL/time point), relative to intensity of β-actin for each time point post PGF_2α_ treatment. (C) Analysis of apoptotic DNA fragmentation in luteal tissue. Genomic DNA isolated from CL tissues collected from untreated control animals (0 h) and from animals at different time points post PGF_2α_ treatment was subjected to DNA laddering analysis. An image of a nylon membrane visualized using PhosphorImager containing the [α^32^P] labeled genomic DNA separated previously on 2% agarose gel and transferred on to nylon membrane is represented here. Migration (base pairs) of oligonucleosomes is indicated on the right.
(TIF)

Figure S3 **Classification of differentially expressed genes post 6 h PGF_2α_ administration by Ingenuity Pathway Analysis (IPA).** (A) The pathway analysis indicates that a large number of differentially expressed genes belongs to canonical pathways such as p38 MAP kinase signaling, VEGF signaling, transcription factors belonging to steroidogenesis and coagulation system. The orange line represents score for the likelihood [-log (B-H P < 0.05)] that genes belonging to a specific canonical pathway category affected at 6 h post PGF_2α_ administration. The stacked bars indicate the percentage of genes distributed according to regulation, i.e., green (down), red (up) and open bars (no overlap with dataset) in each canonical pathway. (B) Network 0 vs. 6 h: Ingenuity Pathway Analysis of the differentially regulated genes 6 h post PGF_2α_ administration shows a network of 28 focus molecules with a score of 44, with top biological functions of cell to cell signaling, molecular transport and lipid metabolism. The network is displayed graphically as nodes (genes/gene products) and edges (biological relationship between nodes). The node color intensity indicates the fold change expression of genes; with red representing up regulation, and green down regulation of genes between 0 vs. 6 h post PGF_2α_ administration. The fold change value for individual gene is indicated under each node. The shapes of nodes indicate the functional class of the gene product and the lines indicate the type of interaction.
(TIF)

Figure S4 **Classification of differentially expressed genes post 18 h PGF_2α_ administration by Ingenuity Pathway Analysis (IPA).** (A) The pathway analysis indicates that a large number of differentially expressed genes belong to canonical pathways such as TGF-β signaling, steroid biosynthesis, NF-κB signaling and coagulation system. The orange line represents score for the likelihood [-log (B-H P<0.05)] that genes belonging to a specific canonical pathway category affected at 18 h post PGF_2α_ administration. The stacked bars indicate the percentage of genes distributed according to regulation, i.e., green (down), red (up) and open bars (no overlap with dataset) in each canonical pathway. (B) Network 0 vs. 18 h: Ingenuity Pathway Analysis of the differentially regulated genes 18 h post PGF_2α_ administration shows a network of 26 focus molecules with a score of 39, with top biological functions of cellular development, cell cycle and gene expression. The network is displayed graphically as nodes (genes/gene products) and edges (biological relationship between nodes). The node color intensity indicates the fold change expression of genes; with red representing up regulation, and green down regulation of genes between 0 vs. 18 h post PGF_2α_ administration. The fold change value for individual gene is indicated under each node. The shapes of nodes indicate the functional class of the gene product and the lines indicate the type of interaction.
(TIF)

Figure S5 **Effect of PGF_2α_ administration on expression of orphan nuclear transcription factors in CL.** (A and B) The orphan nuclear transcription factors associated with regulation of expression of steroidogenic genes were analyzed. Protein levels of NR5A1/SF-1(A) and NR5A2/LRH-1 (B) in bovine CL were determined. Protein lysate (100 µg) prepared from CL tissue collected before (0 h) and post (3, 6 and 18 h) PGF_2α_ treatment were resolved on 10% SDS PAGE, transferred onto PVDF membrane and immunoblot analysis was performed using anti-SF1, anti-LRH1 and anti-β-actin antibody. A representative immunoblot for each of the antibody probed is shown. The immunoblot probed with β-actin antibody indicates loading control for each lane. Densitometric values were determined and indicated as mean±SEM (n = 3 animals/time point), relative to intensity of β-actin for each time point post PGF_2α_ treatment. The values of immunoblot analysis has been put on top of each lane and lane with different letters indicates statistical significance, p<0.05.
(TIF)

Figure S6 **PGF_2α_ administration effect on expression of estrogen receptors during spontaneous and induced luteolysis in CL.** Quantitative real time PCR (qPCR) fold change expression of the estrogen receptors (ERα and ERβ) during spontaneous (A) and induced (B) luteolysis. Total RNA isolated from CL was reverse transcribed and cDNA equivalent to 10 ng of total RNA was used for qPCR. The expression was normalized with L19 mRNA. The results are shown as fold changes of mRNA expression compared with that at early (E) luteal phase (A) and 0 h PGF_2α_ (B) for bovine CL. Individual bar for each gene represents mean±SEM fold change in mRNA expression value for qPCR analysis at each time point (n = 2 animals/time point, A and n = 3 animals/time point, B). For each gene, bars with different letters above them are significantly different (p<0.05).
(TIF)

Table S1 **List of primer set employed for quantitative real time PCR.** The list of genes and details of the primers employed in the qPCR analysis along with the annealing temperature and expected amplicon size are provided.
(TIF)

Table S2 **List of top 15 up regulated genes at 3 h post PGF_2α_ administration.** Microarray data analysis was carried out to obtain a set of differentially expressed genes based on statistics, a Student's t-test (two tail, unpaired) with p<0.05 and multiple hypothesis testing (Benjamini and Hochberg comparison test) to reduce the false positives. The identified differentially expressed genes were transcript consistent and did not hybridize to multiple transcripts, as suggested by the AffyProbeMiner analysis. A Bioconductor analysis was performed with ≥2 fold change as cut-off with statistical filters for identification of differentially expressed genes. Whereas, the top 15 differentially UP regulated genes at 3 h post PGF_2α_ treatment are represented in this table. Probe Set ID: The identifier that refers to a set of probe pairs selected to represent expressed sequences on an array; Fold Change: It is a number describing changes in expression level of a gene compared between control and treatment; Gene ID: Gene symbols extracted from Entrez Gene or UniGene; Gene Title: Gene name extracted from Entrez Gene or UniGene.
(TIF)

Table S3 **List of top 15 down regulated genes at 3 h post PGF_2α_ administration.** The top 15 differentially DOWN regulated genes at 3 h post PGF_2α_ treatment are represented.
(TIF)

Table S4 **List of top 15 up regulated genes at 6 h post PGF_2α_ administration.** The top 15 differentially UP regulated genes at 6 h post PGF_2α_ treatment are represented.
(TIF)

Table S5 **List of top 15 down regulated genes at 6 h post PGF_2α_ administration.** The top 15 differentially DOWN regulated genes at 6 h post PGF_2α_ treatment are represented.
(TIF)

Table S6 **List of top 15 up regulated genes at 18 h post PGF_2α_ administration.** The top 15 differentially UP regulated genes at 18 h post PGF_2α_ treatment are represented.
(TIF)

Table S7 **List of top 15 down regulated genes at 18 h post PGF_2α_ administration.** The top 15 differentially DOWN regulated genes at 18 h post PGF_2α_ treatment are represented.
(TIF)

Table S8 **List of top 15 up regulated E_2_ responsive genes at 3 h post PGF_2α_ administration.** Potential E_2_ responsive genes in bovine CL were identified based on the available list of classical E_2_ responsive genes, genes employed in PCR array human estrogen signaling and the data base, ERGDB. Microarray data analysis was carried out to obtain a set of differentially expressed genes based on statistics, a Student's t-test (two tail, unpaired) with p<0.05 and multiple hypothesis testing (Benjamini and Hochberg comparison test) to reduce the false positives. The identified differentially expressed E_2_ responsive genes were transcript consistent and did not hybridize to multiple transcripts, as suggested by the AffyProbeMiner analysis. A Bioconductor analysis was performed with ≥1 fold change as cut-off and statistical filters for identification of differentially expressed E_2_ responsive genes. Whereas, the top 15 differentially UP regulated genes at 3 h post PGF_2α_ treatment are represented in this table. Probe Set ID: The identifier that refers to a set of probe pairs selected to represent expressed sequences on an array; Fold Change: It is a number describing changes in expression level of a gene compared between control and treatment; Gene ID: Gene symbols extracted from Entrez Gene or UniGene; Gene Title: Gene name extracted from Entrez Gene or UniGene.
(TIF)

Table S9 **List of top 15 down regulated E_2_ responsive genes at 3 h post PGF2α administration.** The top 15 differentially DOWN regulated genes at 3 h post PGF_2α_ treatment are represented. The genes are discussed in the results and discussion section.
(TIF)

Table S10 **List of common up regulated E_2_ responsive genes post 3, 6 and 18 h PGF2α administration.** The common differentially UP regulated E_2_ responsive genes (21 genes) before (0 h) and post (3, 6 and 18 h) PGF_2α_ treatment are represented.
(TIF)

Table S11 **List of common down regulated E_2_ responsive genes post 3, 6 and 18 h PGF2α administration.** The common differentially DOWN regulated E_2_ responsive genes (14 genes) before (0 h) and post (3, 6 and 18 h) PGF_2α_ treatment are represented.
(TIF)

Table S12 **List of common up regulated E_2_ responsive genes in monkey (24 h) and bovine (3 h) CL.** The previously published microarray data of the differentially expressed genes from the CL tissues of macaques receiving PGF_2α_ treatment for 24 h [GEO accession number GSE8371] was mined for E_2_ responsive genes for purposes of comparing the number of E_2_ responsive genes that were differentially expressed in CL of macaques to that of the buffalo cow CL [GEO accession number GSE27961]. The mined data comprising common UP regulated E_2_ responsive genes (19 genes) of macaques CL at 24 h vs. E_2_ responsive genes of bovine CL at 3 h post PGF_2α_ treatment are represented in this Table.
(TIF)

Table S13 **List of common down regulated E_2_ responsive genes in monkey (24 h) and bovine (3 h) CL.** The mined data comprising common DOWN regulated E_2_ responsive genes (8 genes) of macaques CL at 24 h vs. E_2_ responsive genes of bovine CL at 3 h post PGF_2α_ treatment are represented in this Table.
(TIF)

Table S14 **List of common up regulated E_2_ responsive genes in monkey (24 h) and bovine (6 h) CL.** The mined data comprising common UP regulated E_2_ responsive genes (17 genes) of macaques CL at 24 h vs. E_2_ responsive genes of bovine CL at 6 h post PGF_2α_ treatment are represented in this Table.
(TIF)

Table S15 **List of common down regulated E_2_ responsive genes in monkey (24 h) and bovine (6 h) CL.** The mined data comprising common DOWN regulated E_2_ responsive genes (13 genes) of macaques CL at 24 h vs. E_2_ responsive genes of bovine CL at 6 h post PGF_2α_ treatment are represented in this Table.
(TIF)

Table S16 **List of common up regulated E_2_ responsive genes in monkey (24 h) and bovine (18 h) CL.** The mined data comprising common UP regulated E_2_ responsive genes (17 genes) of macaques CL at 24 h vs. E_2_ responsive genes of bovine CL at 18 h post PGF_2α_ treatment are represented in this Table.
(TIF)

Table S17 **List of common down regulated E_2_ responsive genes in monkey (24 h) and bovine (18 h) CL.** The mined data comprising common DOWN regulated E_2_ responsive genes (13 genes) of macaques CL at 24 h vs. E_2_ responsive genes of bovine CL at 6 h post PGF_2α_ treatment are represented in this Table.
(TIF)
