# Supplementary figures and images for: Profiling of Luteal Transcriptome during Prostaglandin F2-Alpha Treatment in Buffalo Cows: Analysis of Signaling Pathways Associated with Luteolysis
Source: PLoS One. 2014 Aug 7;9(8):e104127. doi: 10.1371/journal.pone.0104127 (PMC4125180; doi:10.1371/journal.pone.0104127)

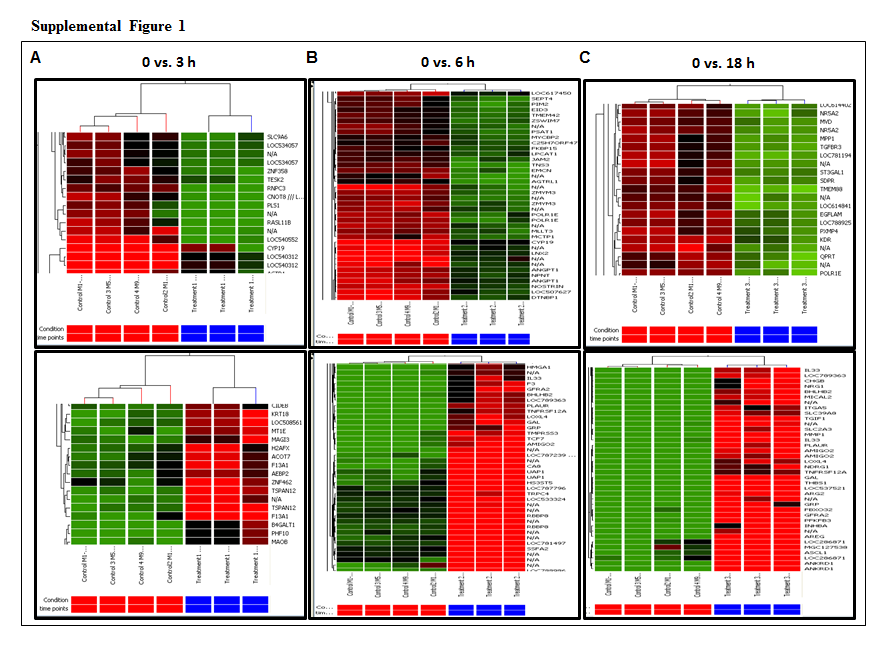

Supplement: Figure S1 — Expanded tree view generated after hierarchical clustering of representative genes obtained after pairwise analysis. The expanded tree displaying the hierarchy analysis for probe sets at each time point [0 vs. 3 h (A), 0 vs. 6 h (B) and 0 vs. 18 h (C)] post PGF2α administration. The bottom of each dendrogram shows the condition color bar with the parameters in each interpretation. The legend shows the name of each condition on which clustering was performed. Header of heat map shows a normalized intensity values represented in various shades of red and green indicating relatively either up or down regulation, respectively. The row header shown on the right side represents the complete entity name or gene symbol. The upper panel shows groups of genes, whose expression was up regulated at 0 h and with the administration of PGF2α the expression is observed to be down-regulated. The lower panel shows groups of genes whose expression was down regulated at 0 h and with the administration of PGF2α the expression is observed to be up regulated. (TIF) [file pone.0104127.s001.tif]

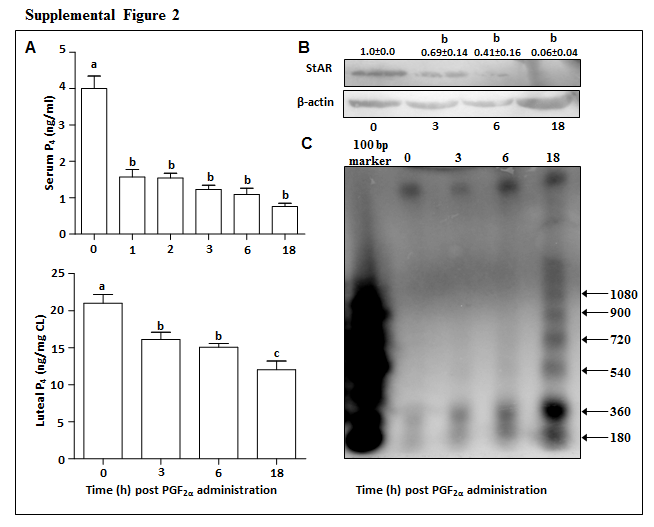

Supplement: Figure S2 — Effects of PGF2α on circulating and luteal P4 levels, StAR expression and DNA fragmentation. Buffalo cows received intramuscular injection of 500 µg of PGF2α on day 11 of estrous cycle and blood and luteal tissue samples at different time intervals post PGF2α treatment. (A) Circulating mean±SEM serum and luteal progesterone (P4) concentrations immediately before (0 h) and at different time points post PGF2α treatment. Bars with different alphabets indicate statistical significance, p<0.05. (B) Protein lysate (100 µg) prepared from CL tissue collected before and post PGF2α treatment were resolved on 10% SDS PAGE, transferred onto PVDF membrane and immunoblot analysis was performed using anti-StAR and anti-β-actin antibody (β-actin was used as loading control). A representative immunoblot for each of the antibody probed is shown. Densitometric values shown on top of each lane represented were determined and represented as mean±SEM (n = 3 CL/time point), relative to intensity of β-actin for each time point post PGF2α treatment. (C) Analysis of apoptotic DNA fragmentation in luteal tissue. Genomic DNA isolated from CL tissues collected from untreated control animals (0 h) and from animals at different time points post PGF2α treatment was subjected to DNA laddering analysis. An image of a nylon membrane visualized using PhosphorImager containing the [α32P] labeled genomic DNA separated previously on 2% agarose gel and transferred on to nylon membrane is represented here. Migration (base pairs) of oligonucleosomes is indicated on the right. (TIF) [file pone.0104127.s002.tif]

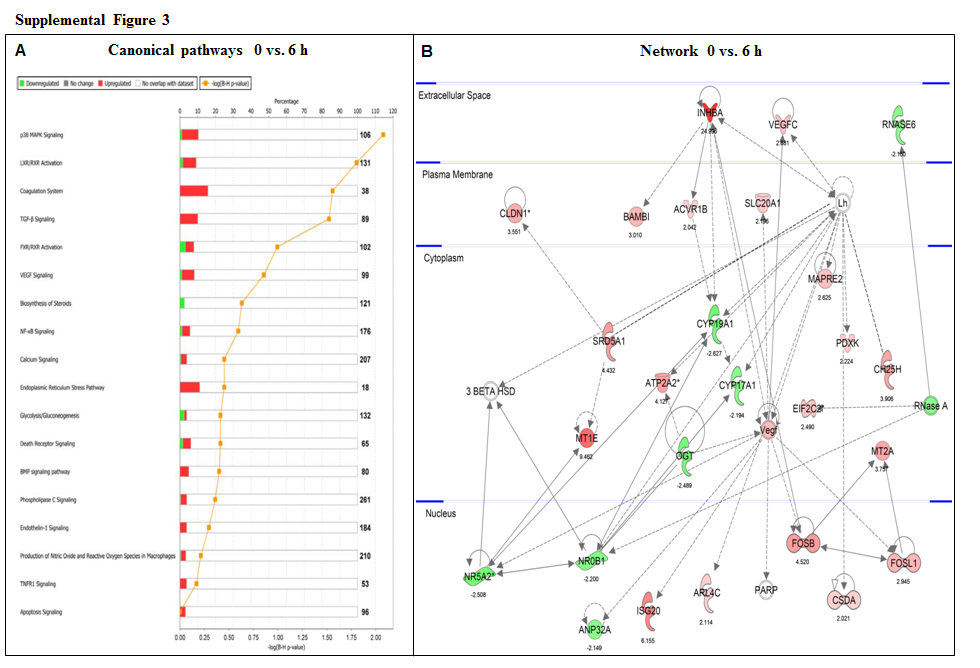

Supplement: Figure S3 — Classification of differentially expressed genes post 6 h PGF2α administration by Ingenuity Pathway Analysis (IPA). (A) The pathway analysis indicates that a large number of differentially expressed genes belongs to canonical pathways such as p38 MAP kinase signaling, VEGF signaling, transcription factors belonging to steroidogenesis and coagulation system. The orange line represents score for the likelihood [-log (B-H P < 0.05)] that genes belonging to a specific canonical pathway category affected at 6 h post PGF2α administration. The stacked bars indicate the percentage of genes distributed according to regulation, i.e., green (down), red (up) and open bars (no overlap with dataset) in each canonical pathway. (B) Network 0 vs. 6 h: Ingenuity Pathway Analysis of the differentially regulated genes 6 h post PGF2α administration shows a network of 28 focus molecules with a score of 44, with top biological functions of cell to cell signaling, molecular transport and lipid metabolism. The network is displayed graphically as nodes (genes/gene products) and edges (biological relationship between nodes). The node color intensity indicates the fold change expression of genes; with red representing up regulation, and green down regulation of genes between 0 vs. 6 h post PGF2α administration. The fold change value for individual gene is indicated under each node. The shapes of nodes indicate the functional class of the gene product and the lines indicate the type of interaction. (TIF) [file pone.0104127.s003.tif]

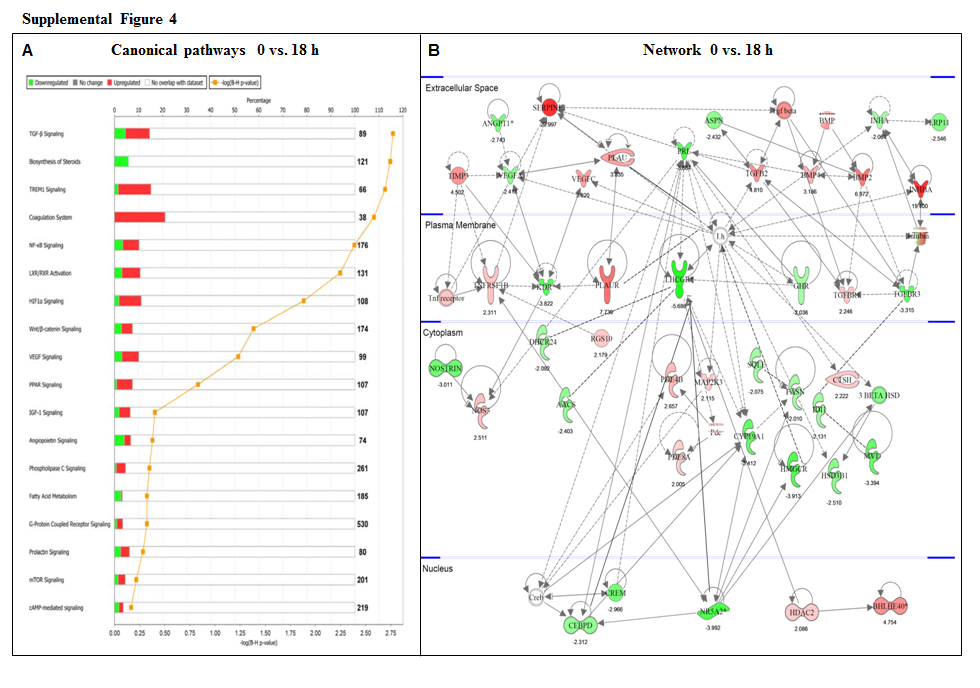

Supplement: Figure S4 — Classification of differentially expressed genes post 18 h PGF2α administration by Ingenuity Pathway Analysis (IPA). (A) The pathway analysis indicates that a large number of differentially expressed genes belong to canonical pathways such as TGF-β signaling, steroid biosynthesis, NF-κB signaling and coagulation system. The orange line represents score for the likelihood [-log (B-H P<0.05)] that genes belonging to a specific canonical pathway category affected at 18 h post PGF2α administration. The stacked bars indicate the percentage of genes distributed according to regulation, i.e., green (down), red (up) and open bars (no overlap with dataset) in each canonical pathway. (B) Network 0 vs. 18 h: Ingenuity Pathway Analysis of the differentially regulated genes 18 h post PGF2α administration shows a network of 26 focus molecules with a score of 39, with top biological functions of cellular development, cell cycle and gene expression. The network is displayed graphically as nodes (genes/gene products) and edges (biological relationship between nodes). The node color intensity indicates the fold change expression of genes; with red representing up regulation, and green down regulation of genes between 0 vs. 18 h post PGF2α administration. The fold change value for individual gene is indicated under each node. The shapes of nodes indicate the functional class of the gene product and the lines indicate the type of interaction. (TIF) [file pone.0104127.s004.tif]

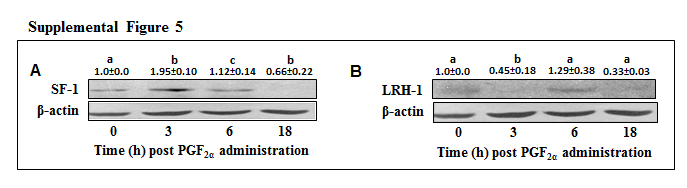

Supplement: Figure S5 — Effect of PGF2α administration on expression of orphan nuclear transcription factors in CL. (A and B) The orphan nuclear transcription factors associated with regulation of expression of steroidogenic genes were analyzed. Protein levels of NR5A1/SF-1(A) and NR5A2/LRH-1 (B) in bovine CL were determined. Protein lysate (100 µg) prepared from CL tissue collected before (0 h) and post (3, 6 and 18 h) PGF2α treatment were resolved on 10% SDS PAGE, transferred onto PVDF membrane and immunoblot analysis was performed using anti-SF1, anti-LRH1 and anti-β-actin antibody. A representative immunoblot for each of the antibody probed is shown. The immunoblot probed with β-actin antibody indicates loading control for each lane. Densitometric values were determined and indicated as mean±SEM (n = 3 animals/time point), relative to intensity of β-actin for each time point post PGF2α treatment. The values of immunoblot analysis has been put on top of each lane and lane with different letters indicates statistical significance, p<0.05. (TIF) [file pone.0104127.s005.tif]

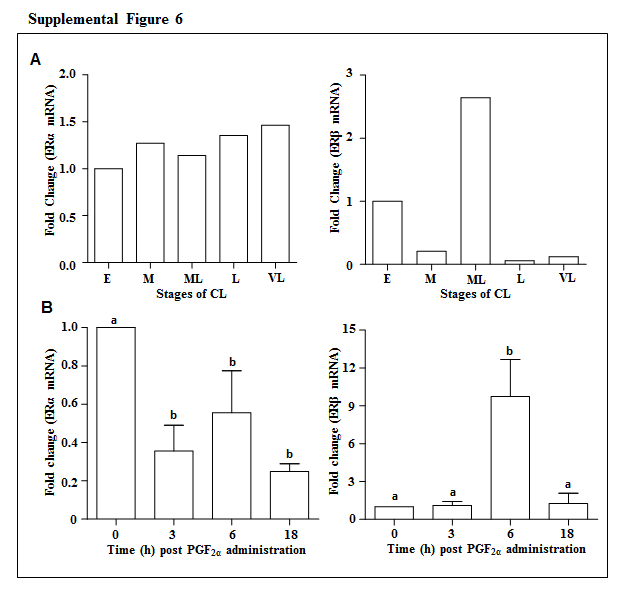

Supplement: Figure S6 — PGF2α administration effect on expression of estrogen receptors during spontaneous and induced luteolysis in CL. Quantitative real time PCR (qPCR) fold change expression of the estrogen receptors (ERα and ERβ) during spontaneous (A) and induced (B) luteolysis. Total RNA isolated from CL was reverse transcribed and cDNA equivalent to 10 ng of total RNA was used for qPCR. The expression was normalized with L19 mRNA. The results are shown as fold changes of mRNA expression compared with that at early (E) luteal phase (A) and 0 h PGF2α (B) for bovine CL. Individual bar for each gene represents mean±SEM fold change in mRNA expression value for qPCR analysis at each time point (n = 2 animals/time point, A and n = 3 animals/time point, B). For each gene, bars with different letters above them are significantly different (p<0.05). (TIF) [file pone.0104127.s006.tif]

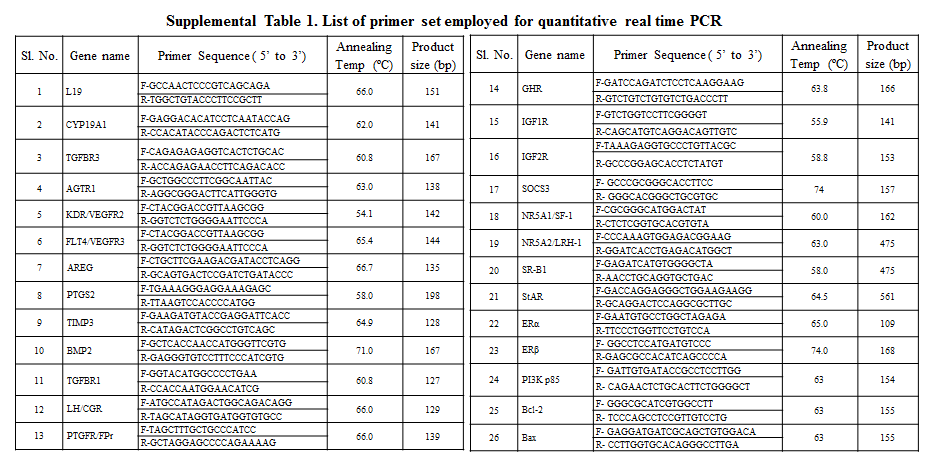

Supplement: Table S1 — List of primer set employed for quantitative real time PCR. The list of genes and details of the primers employed in the qPCR analysis along with the annealing temperature and expected amplicon size are provided. (TIF) [file pone.0104127.s007.tif]

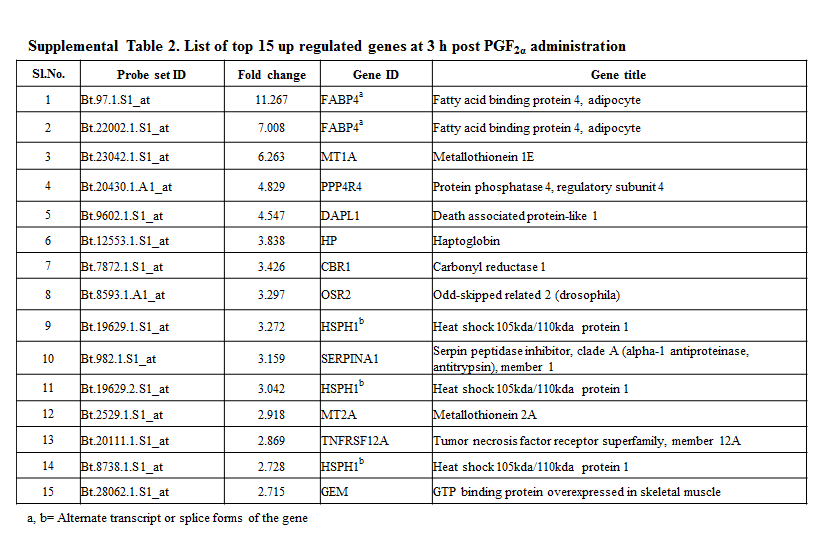

Supplement: Table S2 — List of top 15 up regulated genes at 3 h post PGF2α administration. Microarray data analysis was carried out to obtain a set of differentially expressed genes based on statistics, a Student's t-test (two tail, unpaired) with p<0.05 and multiple hypothesis testing (Benjamini and Hochberg comparison test) to reduce the false positives. The identified differentially expressed genes were transcript consistent and did not hybridize to multiple transcripts, as suggested by the AffyProbeMiner analysis. A Bioconductor analysis was performed with ≥2 fold change as cut-off with statistical filters for identification of differentially expressed genes. Whereas, the top 15 differentially UP regulated genes at 3 h post PGF2α treatment are represented in this table. Probe Set ID: The identifier that refers to a set of probe pairs selected to represent expressed sequences on an array; Fold Change: It is a number describing changes in expression level of a gene compared between control and treatment; Gene ID: Gene symbols extracted from Entrez Gene or UniGene; Gene Title: Gene name extracted from Entrez Gene or UniGene. (TIF) [file pone.0104127.s008.tif]

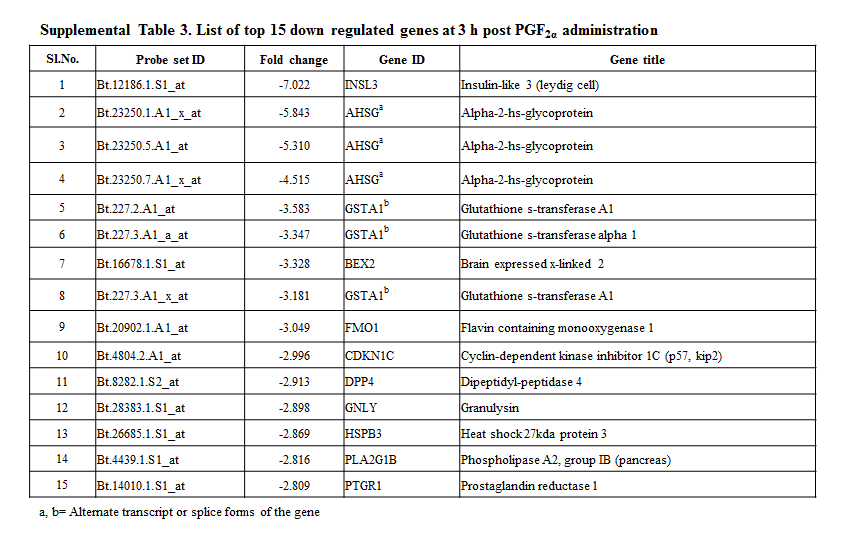

Supplement: Table S3 — List of top 15 down regulated genes at 3 h post PGF2α administration. The top 15 differentially DOWN regulated genes at 3 h post PGF2α treatment are represented. (TIF) [file pone.0104127.s009.tif]

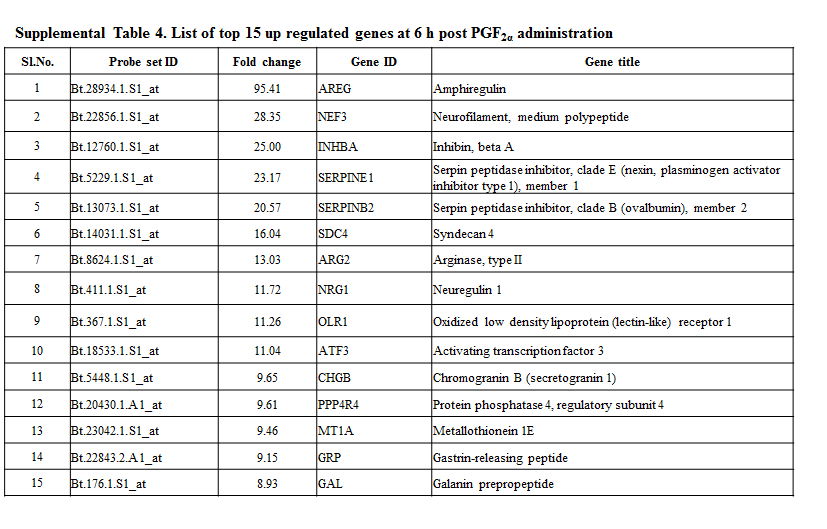

Supplement: Table S4 — List of top 15 up regulated genes at 6 h post PGF2α administration. The top 15 differentially UP regulated genes at 6 h post PGF2α treatment are represented. (TIF) [file pone.0104127.s010.tif]

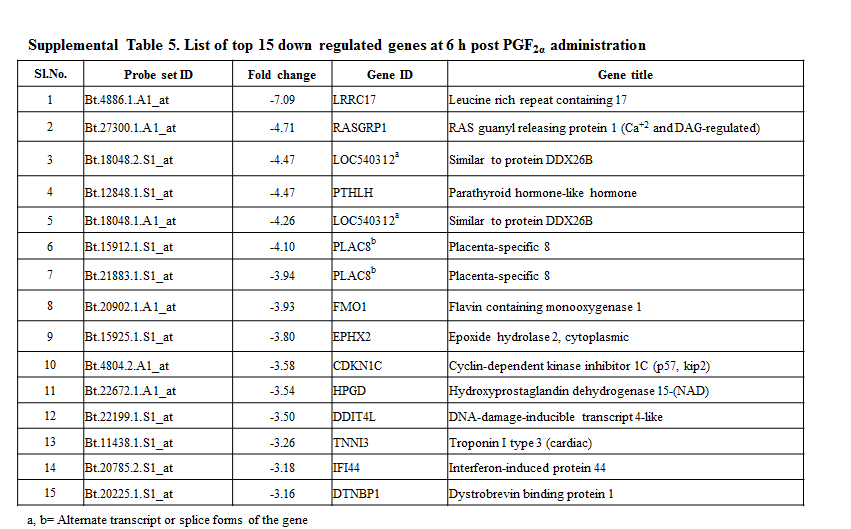

Supplement: Table S5 — List of top 15 down regulated genes at 6 h post PGF2α administration. The top 15 differentially DOWN regulated genes at 6 h post PGF2α treatment are represented. (TIF) [file pone.0104127.s011.tif]

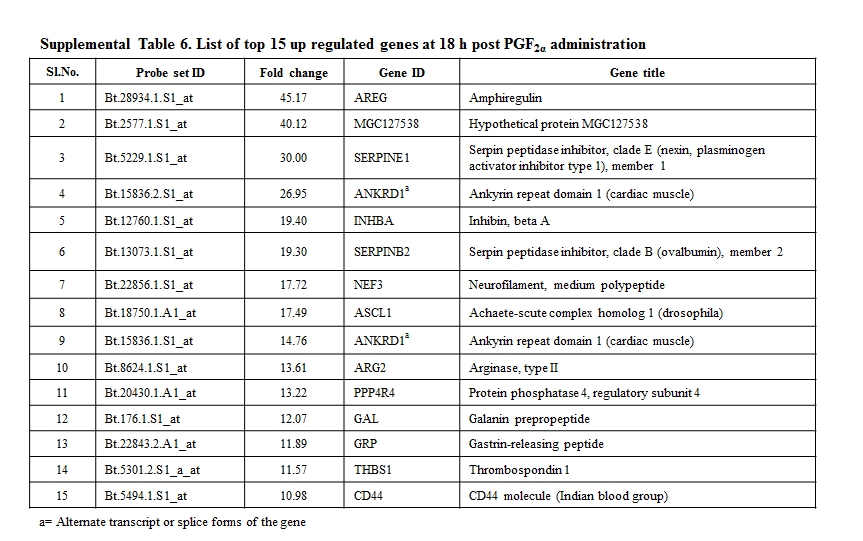

Supplement: Table S6 — List of top 15 up regulated genes at 18 h post PGF2α administration. The top 15 differentially UP regulated genes at 18 h post PGF2α treatment are represented. (TIF) [file pone.0104127.s012.tif]

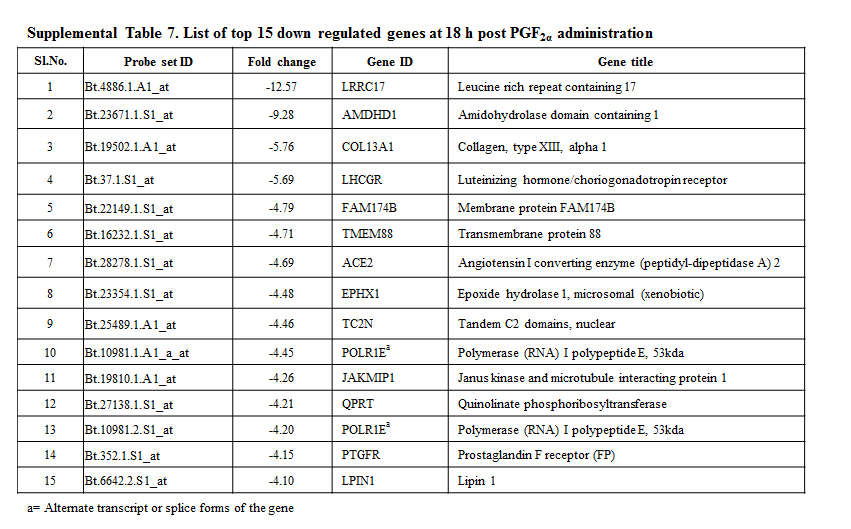

Supplement: Table S7 — List of top 15 down regulated genes at 18 h post PGF2α administration. The top 15 differentially DOWN regulated genes at 18 h post PGF2α treatment are represented. (TIF) [file pone.0104127.s013.tif]

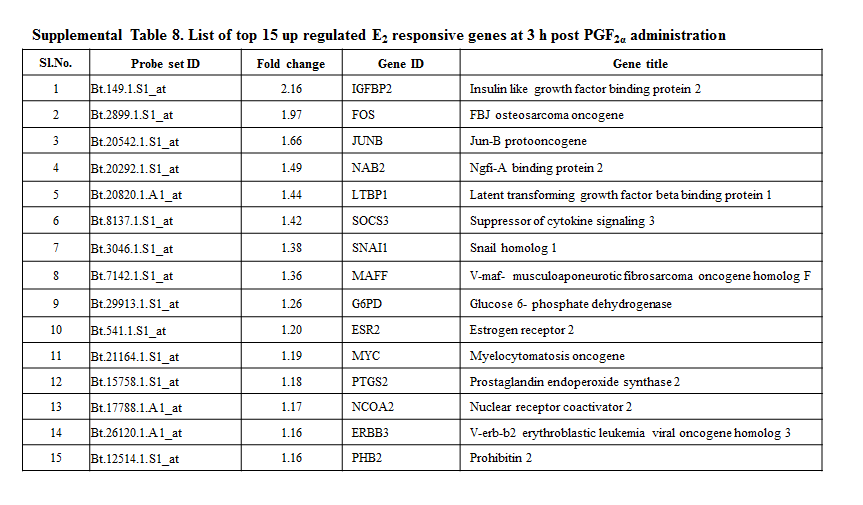

Supplement: Table S8 — List of top 15 up regulated E2 responsive genes at 3 h post PGF2α administration. Potential E2 responsive genes in bovine CL were identified based on the available list of classical E2 responsive genes, genes employed in PCR array human estrogen signaling and the data base, ERGDB. Microarray data analysis was carried out to obtain a set of differentially expressed genes based on statistics, a Student's t-test (two tail, unpaired) with p<0.05 and multiple hypothesis testing (Benjamini and Hochberg comparison test) to reduce the false positives. The identified differentially expressed E2 responsive genes were transcript consistent and did not hybridize to multiple transcripts, as suggested by the AffyProbeMiner analysis. A Bioconductor analysis was performed with ≥1 fold change as cut-off and statistical filters for identification of differentially expressed E2 responsive genes. Whereas, the top 15 differentially UP regulated genes at 3 h post PGF2α treatment are represented in this table. Probe Set ID: The identifier that refers to a set of probe pairs selected to represent expressed sequences on an array; Fold Change: It is a number describing changes in expression level of a gene compared between control and treatment; Gene ID: Gene symbols extracted from Entrez Gene or UniGene; Gene Title: Gene name extracted from Entrez Gene or UniGene. (TIF) [file pone.0104127.s014.tif]

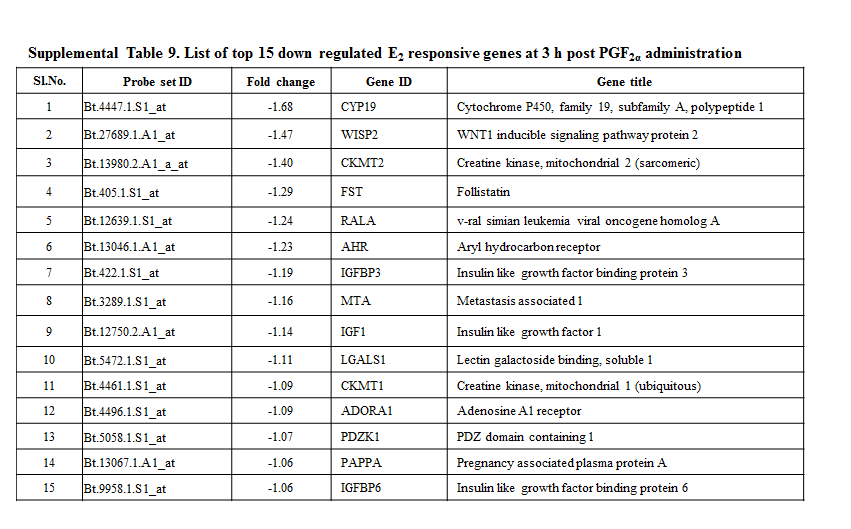

Supplement: Table S9 — List of top 15 down regulated E2 responsive genes at 3 h post PGF2α administration. The top 15 differentially DOWN regulated genes at 3 h post PGF2α treatment are represented. The genes are discussed in the results and discussion section. (TIF) [file pone.0104127.s015.tif]

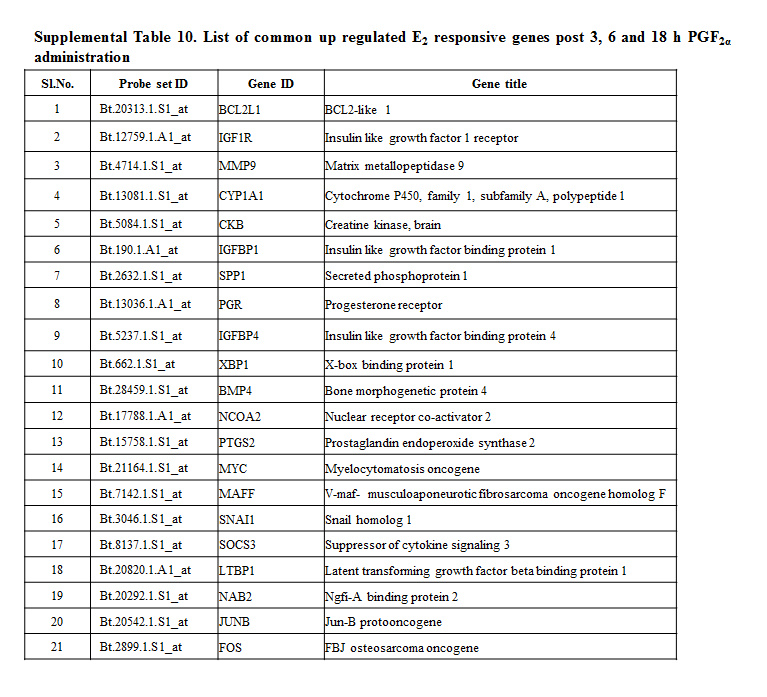

Supplement: Table S10 — List of common up regulated E2 responsive genes post 3, 6 and 18 h PGF2α administration. The common differentially UP regulated E2 responsive genes (21 genes) before (0 h) and post (3, 6 and 18 h) PGF2α treatment are represented. (TIF) [file pone.0104127.s016.tif]

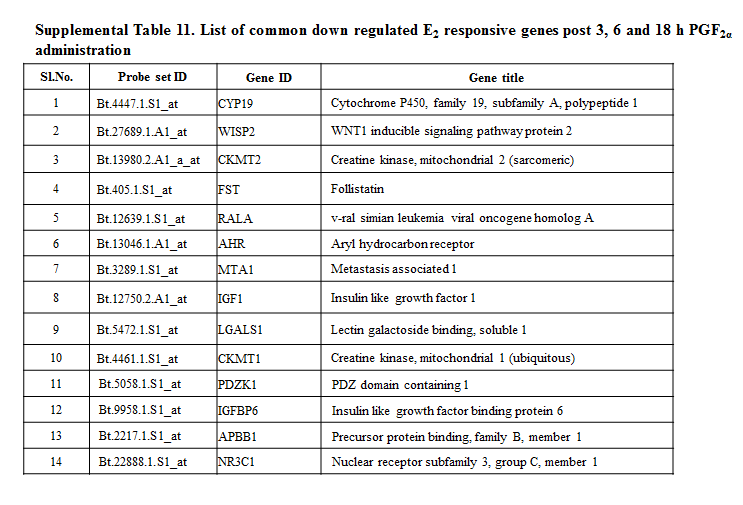

Supplement: Table S11 — List of common down regulated E2 responsive genes post 3, 6 and 18 h PGF2α administration. The common differentially DOWN regulated E2 responsive genes (14 genes) before (0 h) and post (3, 6 and 18 h) PGF2α treatment are represented. (TIF) [file pone.0104127.s017.tif]

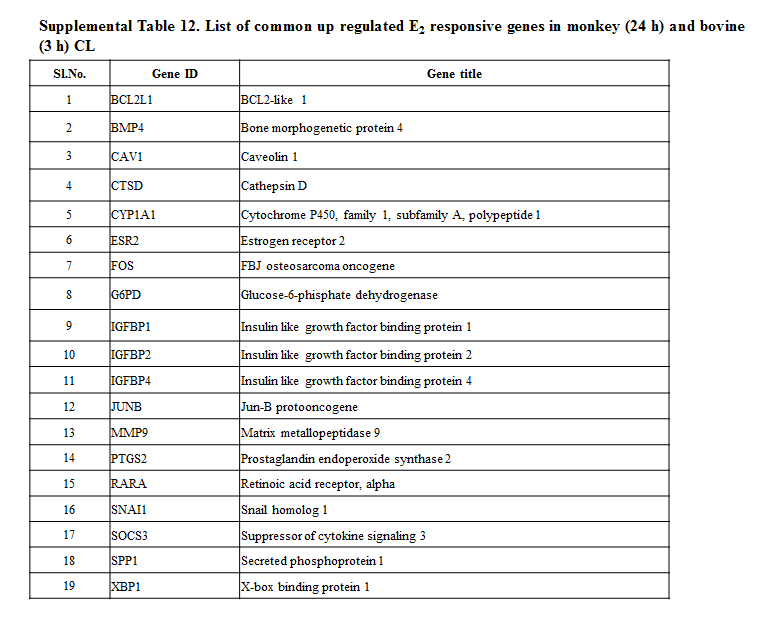

Supplement: Table S12 — List of common up regulated E2 responsive genes in monkey (24 h) and bovine (3 h) CL. The previously published microarray data of the differentially expressed genes from the CL tissues of macaques receiving PGF2α treatment for 24 h [GEO accession number GSE8371] was mined for E2 responsive genes for purposes of comparing the number of E2 responsive genes that were differentially expressed in CL of macaques to that of the buffalo cow CL [GEO accession number GSE27961]. The mined data comprising common UP regulated E2 responsive genes (19 genes) of macaques CL at 24 h vs. E2 responsive genes of bovine CL at 3 h post PGF2α treatment are represented in this Table. (TIF) [file pone.0104127.s018.tif]

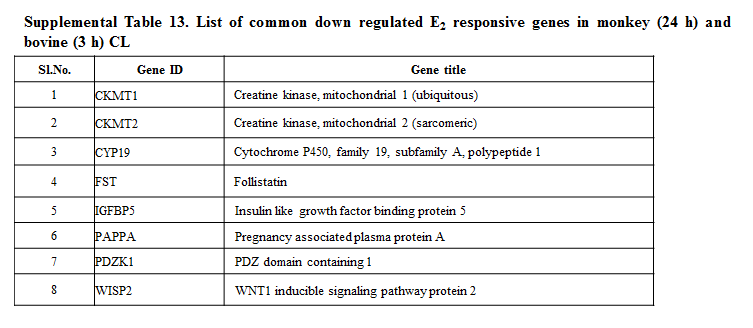

Supplement: Table S13 — List of common down regulated E2 responsive genes in monkey (24 h) and bovine (3 h) CL. The mined data comprising common DOWN regulated E2 responsive genes (8 genes) of macaques CL at 24 h vs. E2 responsive genes of bovine CL at 3 h post PGF2α treatment are represented in this Table. (TIF) [file pone.0104127.s019.tif]

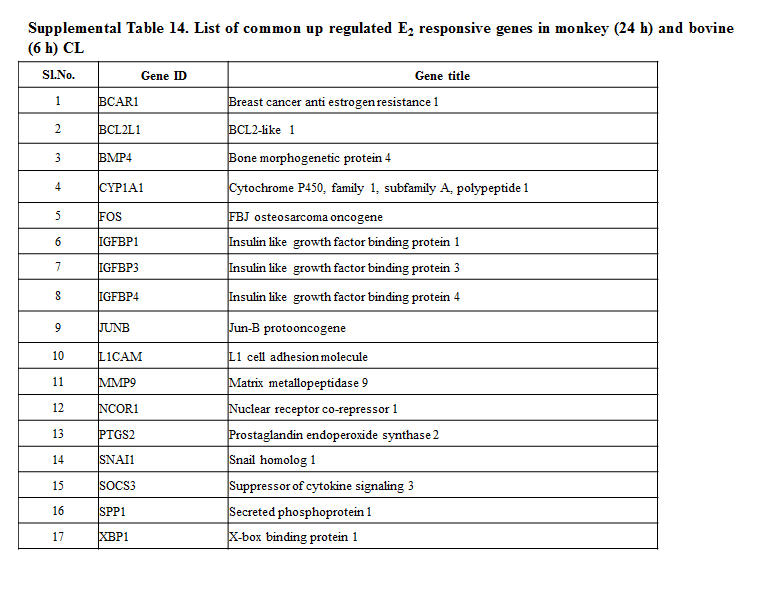

Supplement: Table S14 — List of common up regulated E2 responsive genes in monkey (24 h) and bovine (6 h) CL. The mined data comprising common UP regulated E2 responsive genes (17 genes) of macaques CL at 24 h vs. E2 responsive genes of bovine CL at 6 h post PGF2α treatment are represented in this Table. (TIF) [file pone.0104127.s020.tif]

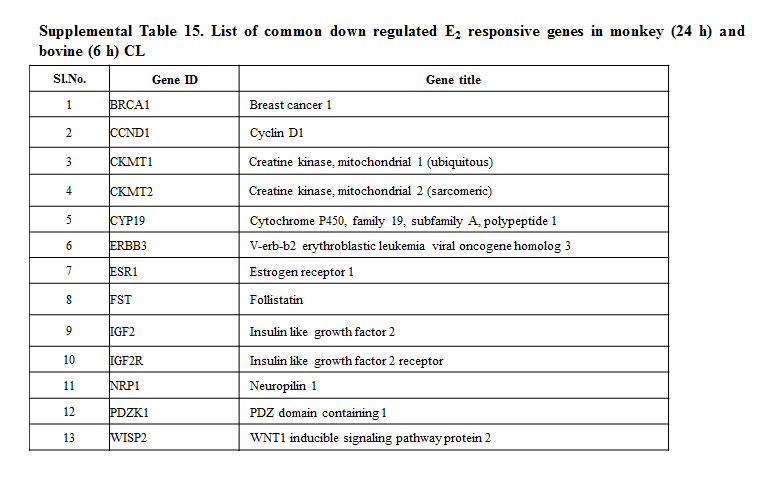

Supplement: Table S15 — List of common down regulated E2 responsive genes in monkey (24 h) and bovine (6 h) CL. The mined data comprising common DOWN regulated E2 responsive genes (13 genes) of macaques CL at 24 h vs. E2 responsive genes of bovine CL at 6 h post PGF2α treatment are represented in this Table. (TIF) [file pone.0104127.s021.tif]

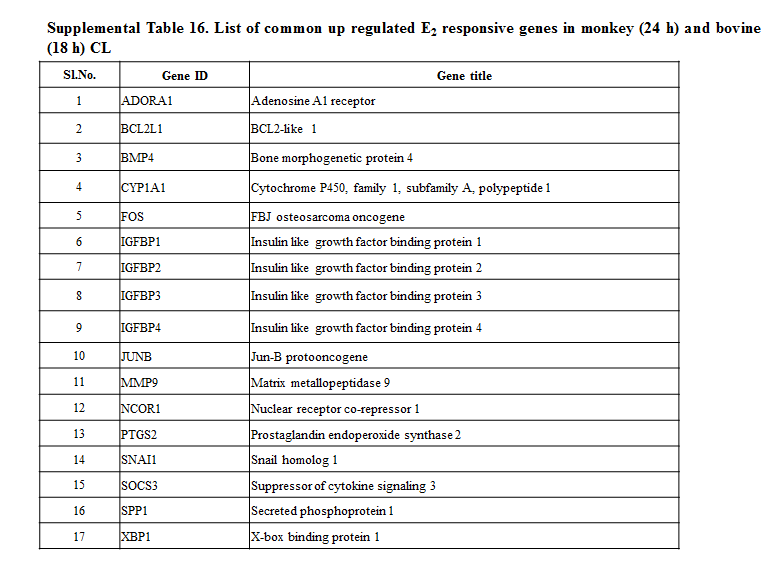

Supplement: Table S16 — List of common up regulated E2 responsive genes in monkey (24 h) and bovine (18 h) CL. The mined data comprising common UP regulated E2 responsive genes (17 genes) of macaques CL at 24 h vs. E2 responsive genes of bovine CL at 18 h post PGF2α treatment are represented in this Table. (TIF) [file pone.0104127.s022.tif]

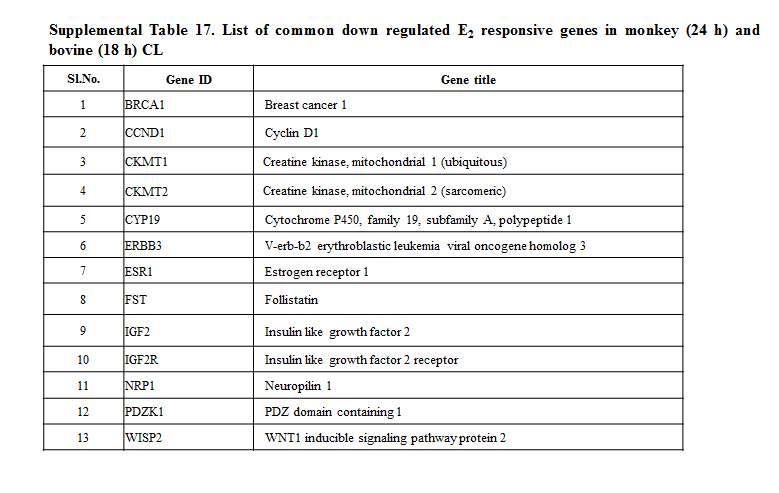

Supplement: Table S17 — List of common down regulated E2 responsive genes in monkey (24 h) and bovine (18 h) CL. The mined data comprising common DOWN regulated E2 responsive genes (13 genes) of macaques CL at 24 h vs. E2 responsive genes of bovine CL at 6 h post PGF2α treatment are represented in this Table. (TIF) [file pone.0104127.s023.tif]
